# Specificity and mechanism of TonB-dependent ferric catecholate uptake by Fiu

**DOI:** 10.3389/fmicb.2024.1355253

**Published:** 2024-03-27

**Authors:** Taihao Yang, Ye Zou, Ho Leung Ng, Ashish Kumar, Salete M. Newton, Phillip E. Klebba

**Affiliations:** Department of Biochemistry and Molecular Biophysics, Kansas State University, Manhattan, KS, United States

**Keywords:** siderophore, catecholate, iron transport, TonB, fluorescent sensor, site-directed mutagenesis, bacterial pathogenesis, Trojan horse antibiotic

## Abstract

We studied the *Escherichia coli* outer membrane protein Fiu, a presumed transporter of monomeric ferric catecholates, by introducing Cys residues in its surface loops and modifying them with fluorescein maleimide (FM). Fiu-FM bound iron complexes of the tricatecholate siderophore enterobactin (FeEnt) and glucosylated enterobactin (FeGEnt), their dicatecholate degradation product Fe(DHBS)_2_ (FeEnt*), the monocatecholates dihydroxybenzoic acid (FeDHBA) and dihydroxybenzoyl serine (FeDHBS), and the siderophore antibiotics cefiderocol (FDC) and MB-1. Unlike high-affinity ligand-gated porins (LGPs), Fiu-FM had only micromolar affinity for iron complexes. Its apparent K_D_ values for FeDHBS, FeDHBA, FeEnt*, FeEnt, FeGEnt, FeFDC, and FeMB-1 were 0.1, 0.7, 0.7, 1.0, 0.3, 0.4, and 4 μM, respectively. Despite its broad binding abilities, the transport repertoires of *E. coli* Fiu, as well as those of Cir and FepA, were less broad. Fiu only transported FeEnt*. Cir transported FeEnt* and FeDHBS (weakly); FepA transported FeEnt, FeEnt*, and FeDHBA. Both Cir and FepA bound FeGEnt, albeit with lower affinity. Related transporters of *Acinetobacter baumannii* (PiuA, PirA, BauA) had similarly moderate affinity and broad specificity for di- or monomeric ferric catecholates. Both microbiological and radioisotopic experiments showed Fiu’s exclusive transport of FeEnt*, rather than ferric monocatecholate compounds. Molecular docking and molecular dynamics simulations predicted three binding sites for FeEnt*in the external vestibule of Fiu, and a fourth site deeper in its interior. Alanine scanning mutagenesis in the outermost sites (1a, 1b, and 2) decreased FeEnt* binding affinity as much as 20-fold and reduced or eliminated FeEnt* uptake. Finally, the molecular dynamics simulations suggested a pathway of FeEnt* movement through Fiu that may generally describe the process of metal transport by TonB-dependent receptors.

## Introduction

With few exceptions, terrestrial organisms require iron for growth, but the limited solubility of the predominant aqueous form of iron, ferric oxyhydroxide [Fe(OH)_n;_ K_SP_ = 2.8 × 10^−39^; Neilands, 1974], obstructs its biological utilization. Consequently, microorganisms produce siderophores (Neilands, 1995) that solubilize Fe^3+^ from its insoluble precipitates, liberating the metal for uptake and incorporation into cellular metabolism. The tricatecholate Gram (−) bacterial siderophore enterobactin (Ent) is an avid iron chelator [K_A_ = 10^52^ M^−1^; Harris et al., 1979]. Some pathogenic bacteria glucosylate Ent (GEnt; also called salmochelin Hantke et al., 2003) to evade siderocalin (Doneanu et al., 2004; Holmes et al., 2005), an innate immune system protein that tightly binds Ent (but not GEnt) (Kumar et al., 2022) and removes it from circulation. The phenolic chelation groups of Ent and GEnt may oxidize to quinones, and their trilactone scaffolds may hydrolyze to yield mono- and dicatecholates (Ent*). In response to these phenomena, Gram (−) bacteria may utilize both FeEnt and FeGEnt, their dicatecholate hydrolysis product ferric (dihydroxybenzolyserine)_2_ (Fe(DHBS)_2_; FeEnt*), and monocatecholates (dihydroxybenzoic acid (FeDHBA_3_); FeDHBS_3_). The *E. coli* cell envelope transport systems for ferric catecholates encompass outer membrane (OM) receptors (Fiu, FepA, Cir, IroN Klebba et al., 2021), periplasmic binding proteins (FepB Thulasiraman et al., 1998; Sprencel et al., 2000), inner membrane (IM) ABC-transporters FepCDG (Shea and McIntosh, 1991), and Ent hydrolases/membrane reductase activities (Fes, IroD, IroE) (Brickman and McIntosh, 1992; Lin et al., 2005; Zhu et al., 2005; Caza et al., 2015). Monocatecholate Trojan horse siderophore antibiotics (Budzikiewicz, 2001; Wencewicz et al., 2013; Simner and Patel, 2020) enter bacterial cells by the same pathways.

Gram (−) bacterial OM receptors for metal complexes are also called TonB-dependent transporters [TBDT; (Schauer et al., 2008)] or ligand-gated porins [LGPs; (Rutz et al., 1992)]. The initial LGP crystal structures of FepA (Buchanan et al., 1999) and FhuA (Locher et al., 1998) revealed C-terminal, ~650 a.a., 22-stranded, transmembrane β-barrels filled by N-terminal, ~150-a.a. globular domains (Locher et al., 1998; Buchanan et al., 1999; Ferguson et al., 2002; Shultis et al., 2006; Buchanan et al., 2007; Grinter and Lithgow, 2019a). The external loops of LGP β-barrels initiate iron acquisition (Smallwood et al., 2014) by binding particular iron complexes (Klebba et al., 2021; Kumar et al., 2022). The internal N-domain controls the ensuing TonB-dependent stage of ligand passage into the periplasm. TonB (Noinaj et al., 2010) is a cell envelope protein that facilitates active OM iron transport, through connection with electrochemical proton motive force (PMF) across the IM (Bradbeer, 1993; Jordan et al., 2013). TonB anchors in the IM but spans the periplasm to interact with ligand-bound LGP at the periplasmic interface of the OM. Overall, LGPs use PMF, mediated by TonB, to transport ferric siderophores through the OM (Jordan et al., 2013). The structural features of LGP raised the possibility of ligand transport by a “Ball and Chain” mechanism (Armstrong and Bezanilla, 1977), in which the N-terminus dislodges from the channel into the periplasm (Ma et al., 2007). Molecular dynamics simulations suggested that this process may occur by unfolding of the N-terminal globular domain (Faraldo-Gómez et al., 2003; Gumbart et al., 2007), but subsequent experiments concluded that the N-domain of FepA remains within the transmembrane channel and undergoes conformational rearrangements during FeEnt uptake (Majumdar et al., 2020). At present, the molecular mechanism of ferric siderophore movement through OM LGP is an open question.

High-affinity ferric siderophore uptake by TonB-dependent LGP enables Gram-negative bacterial iron acquisition in iron-deficient environments, including within human and animal organs, tissues, fluids, and secretions, where transferrin, siderocalin, and ferritin sequester iron as a defense mechanism (Cornelissen and Sparling, 1994; Abergel et al., 2008; Correnti and Strong, 2012). Consequently, siderophore biosynthetic systems and TonB-dependent ferric siderophore transport systems are determinants of bacterial pathogenesis, including in the ESKAPE organisms (Russo et al., 2002, 2011, 2015): disruption of iron uptake reduces virulence. In *Acinetobacter baumannii*, for example, iron acquisition strongly correlates with bacterial pathogenesis (Gaddy et al., 2012; Penwell et al., 2015; Fleming et al., 2017; Luna et al., 2019; Ramirez et al., 2019; Escalante et al., 2023). Furthermore, orthologs of *E. coli* Fiu and Cir (PiuA and PirA, respectively) were implicated in the uptake of the novel Trojan horse antibiotic cefiderocol (FDC) in both *Pseudomonas aeruginosa* (Luscher et al., 2018; Gupta et al., 2022) and *A. baumannii* (Luscher et al., 2018; Malik et al., 2020; Asrat et al., 2023; Smoke et al., 2023; Tiseo et al., 2023). Siderophore antibiotics use native, TonB-dependent iron uptake pathways for entry into bacterial cells (Miller et al., 1991; Ji et al., 2012; Miller and Liu, 2021).

Fiu [Ferric iron uptake; (Hantke, 1983)] is an LGP (Grinter and Lithgow, 2019a) that was implicated in the uptake of ferric monocatecholates (Curtis et al., 1988; Hantke, 1990; Nikaido and Rosenberg, 1990). It occurs in commensal *E. coli* and in clinically relevant members of Enterobacterales, such as pathogenic *E. coli*, *Klebsiella pneumoniae,* and *A. baumannii* (Klebba et al., 2021). While *E. coli* FepA (EcoFepA) was originally identified as the cognate receptor for FeEnt, Fiu and Cir were proposed to transport ferric monocatecholates, such as FeDHBS and FeDHBA (Hantke, 1990). Other studies showed Fiu-mediated uptake of catecholate siderophore antibiotics (Curtis et al., 1988; Nikaido and Rosenberg, 1990). However, previous research did not define Fiu’s ligand preferences, binding affinities, nor the mechanism by which it captures and internalizes ligands. In this report, we characterized the selectivities of known and putative OM catecholate receptors of *E. coli* (Fiu, EcoFepA, and Cir) and *A. baumannii* (PiuA, AbaFepA, PirA, and BauA), for different catecholate iron complexes, using sensitive fluorescent technology to monitor their binding reactions (Hanson et al., 2016; Nairn et al., 2017; Chakravorty et al., 2019; Kumar et al., 2022). Fiu-FM bound FeEnt, FeGEnt, FeEnt*, FeDHBS, FeDHBA, FeFDC, and FeMB-1. The fluorescent constructs defined the hierarchy of Fiu binding affinities among these iron complexes: FeDHBS (K_D_ = ~0.1 μM); FeGEnt (K_D_ = 0.34 μM); FeEnt* (K_D_ = 0.7 μM); FeDHBA (K_D_ = 0.7 μM); FeEnt (K_D_ = 0.83 μM). However, despite its broad binding activity, Fiu only catalyzed the uptake of FeEnt*, the dicatecholate degradation product of FeEnt: Fiu did not transport FeDHBA, FeDHBS, FeEnt, or FeGEnt. Finally, *in silico* docking experiments and molecular dynamic simulations found 3 FeEnt* binding sites in Fiu’s vestibule and a fourth site deeper in its interior. We used Ala scanning mutagenesis to study the outer sites and identified residues in each location that affected the binding and transport of FeEnt*. Double mutations involving R142A in site 2 created a 20-fold decrease in overall binding affinity and abrogated FeEnt* uptake. Overall, the analysis explained the pathways of ferric catecholate uptake in *E. coli* and *A. baumannii* and provided insight into the general mechanism of metal transport through TonB-dependent LGP.

## Results

### Purification and characterization of natural ferric catecholate siderophores

We purified catecholate ferric siderophores from the pathogenic *E. coli* strain CP9. After culturing it to late log phase in iron-deficient T-media (McIntosh and Earhart, 1976), we clarified the spent culture supernatant by centrifugation, added FeCl_3_ to form ferric siderophores, and purified them by ion exchange, gel filtration, and hydrophobic chromatography (Sephadex LH20), which segregated three distinctly colored red or purple fractions (Supplementary Figure S1; Kumar et al., 2022). The third peak was FeEnt, as identified by its absorption maximum at 495 nm, and by mass spectrometry, which showed a peak at 729.6 m/z (Supplementary Figure S1), in close correspondence to the mass of FeEnt (MW = 719). FeEnt is chemically labile: in aqueous buffers, it degrades over days or weeks, even on ice. As FeEnt storage time passed, the amount of peak 3 decreased and peak 2 increased, consistent with peak 2 as a degradation product of FeEnt. Hence, we designated the 2nd LH20 fraction as FeEnt*. It had an absorption maximum at 503 nm, and mass spectrometry defined it as 554.6 m/z, near the mass of Fe(DHBS)_2_ (MW = 518.4). Four catechol oxygens complex iron (III) in dimeric FeEnt*; in the hexacoordinate ferric complex, two more oxygens may derive from water molecules. When included in mass calculations of FeEnt* [i.e., Fe(DHBS)_2_·2H_2_O], the water molecules raised its mass to 554.4, which matched the mass spectrometric peak at 554.6 m/z. We also characterized the recognition of FeGEnt by Fiu. The glucosylated ferric catecholate does not adsorb well to siderocalin (Kumar et al., 2022), which promotes the pathogenesis of clinical isolates of *E. coli, K. pneumoniae,* and *Salmonella typhimurium* in human and animal hosts (Russo et al., 2011, 2015). We purified FeGEnt from *E. coli* CP9. It comprised the rapidly migrating first peak from LH20, with an extinction maximum at 510 nm and an MS value of 1076.3 m/z, consistent with its predicted molecular mass of 1077.5. Although the peak fraction containing FeEnt* was nearly homogeneous, the fractions containing FeEnt and FeGEnt contained other chemical species, slightly different in mass. This result underscores the chemical lability of FeEnt/FeGEnt and/or their susceptibility to cellular degradative processes during biosynthesis (Lin et al., 2005). Nevertheless, the FeEnt peak fractions did not contain FeEnt*, and the FeGEnt peak fractions did not contain FeEnt. Overall, the procedures yielded FeEnt, FeEnt*, and FeGEnt for use in binding and uptake experiments.

### Siderophore nutrition tests of ferric catecholate transport by Fiu

**Figure 1:**
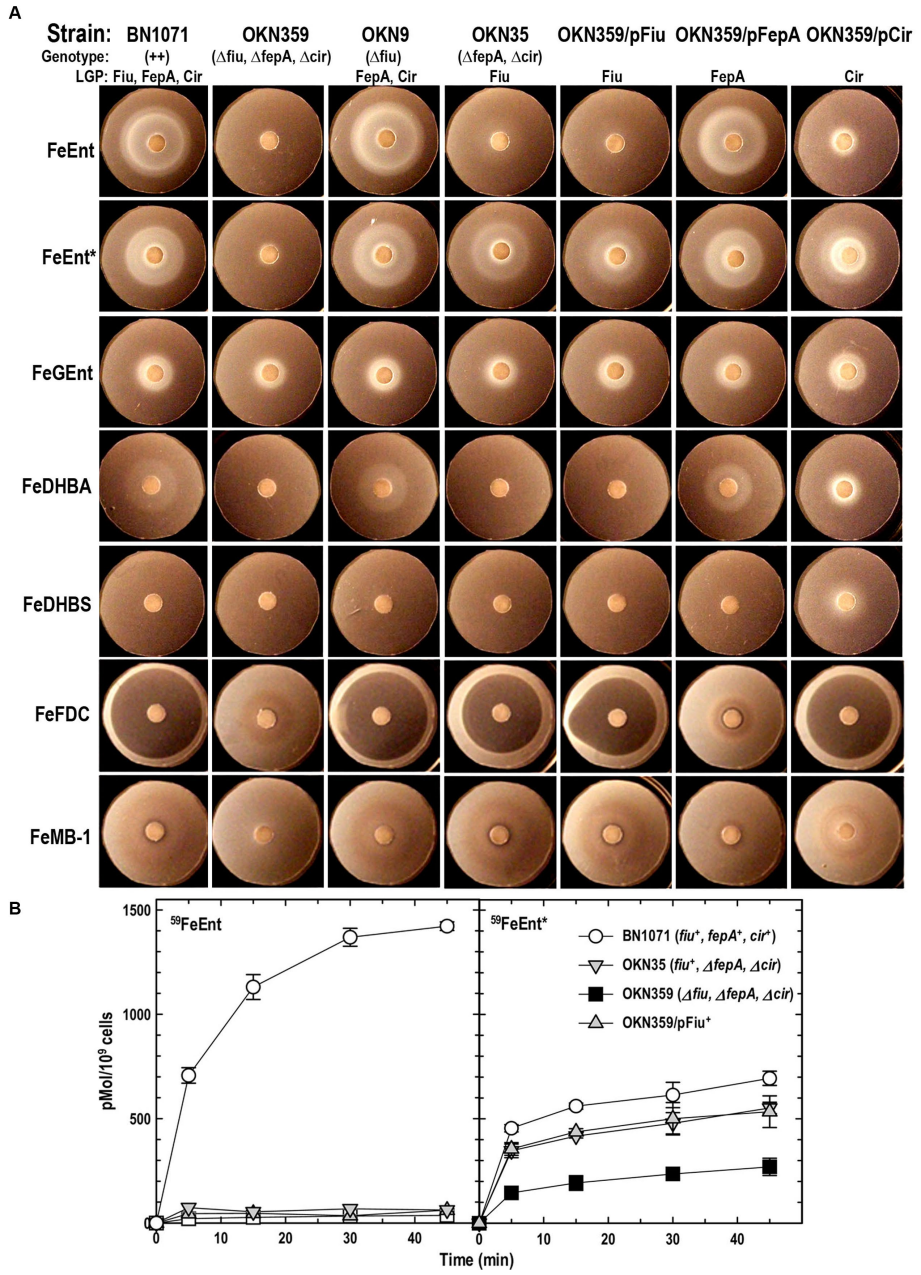
Uptake of ferric catecholates by Fiu, FepA, and Cir. **(A)** Siderophore nutrition tests. We compared strains that selectively expressed Fiu, FepA, and Cir by siderophore nutrition tests (Wayne et al., 1976), and for susceptibility to siderophore antibiotics (FeFDC or FeMB-1), by applying 10 μL of 100 μM solution of test compound to a paper disk on the agar. After overnight incubation at 37°C, we measured the diameter of the growth or killing halos of the siderophore or antibiotic, respectively, with a ruler. **(B)** ^59^Fe accumulation assays. We grew bacteria expressing Fiu, FepA, and Cir, or isogenic derivatives that only expressed Fiu, in MOPS minimal media, added 10 μM [^59^Fe]Ent or [^59^Fe]Ent* at *t* = 0, and collected and filtered aliquots at sequential times. We measured the radioactivity retained on the filters to evaluate the uptake of ferric siderophores. Error bars represent the standard deviations of the means of three independent trials.

Previous reports of ferric monocatecholate uptake by Fiu (Hantke, 1990; Nikaido and Rosenberg, 1990) suggested that it may transport the dicatecholate degradation product, FeEnt*. To test this idea, we compared isogenic chromosomal mutants of BN1071 (Klebba, 1981; Klebba et al., 1982) lacking (Ma et al., 2007) FepA (OKN3: *fiu^+^,* Δ*fepA; cir^+^*), Cir (OKN5; *fiu^+^ fepA^+:^* Δ*cir*), or Fiu (OKN9: Δ*fiu fepA^+^; cir^+^*) to the wild-type and the triple deletion mutant (OKN359: Δ*fiu/*Δ*fepA/*Δ*cir*). We also expressed chromosomal Fiu as sole catecholate-specific LGP (OKN35: *fiu^+^* Δ*fepA;* Δ*cir*) and from the low-copy vector pHSG575 in OKN359. With these and other test strains, we screened the catecholate ferric siderophores FeEnt, FeEnt*, FeGEnt, FeDHBS, and FeDHBA in nutrition tests (Figure 1A; Table 1) that revealed a variety of uptake pathways. Strains that exclusively expressed Fiu grew around disks containing FeEnt*, with halos that were comparable in size and density to those produced by wild-type *E. coli.* In addition to definitive growth around disks containing FeEnt*, Fiu^+^ strains showed very faint, almost imperceptible growth around FeEnt, but no growth around FeDHBA nor FeDHBS. Cells expressing FepA, on the other hand, acquired FeEnt, FeEnt*, and FeDHBA (confirming that the assay functioned properly with FeDHBA). These data demonstrated the uptake specificity of Fiu for FeEnt* but not the ferric monocatecholates. Bacteria singly expressing Fiu, FepA, or Cir all utilized FeEnt*, revealing three uptake pathways for the ferric dicatecholate degradation product. In summary, Fiu transported FeEnt*, very weakly transported FeEnt, but did not utilize FeDHBS nor FeDHBA. Unfortunately, nutrition tests were uninformative for FeGEnt, which produced identical small halos on all the test strains, including *E. coli* BN1071 (Fiu^+^, FepA^+^, Cir^+^) and OKN359 (Δ*fiu/*Δ*fepA/*Δ*cir*). Hence, the assay did not provide evidence of specific FeGEnt uptake by any of the ferric catecholate LGP. However, the uptake of FeGEnt requires not only an OM receptor but also the *iro* gene cluster, that encodes the enterobactin trilactone hydrolases IroD and IroE (Lin et al., 2005). They are present in *S. enterica* and certain pathogenic *E. coli* such as uropathogenic *E. coli* 563 (Hantke et al., 2003), but the laboratory *E. coli* host strains we employed lack the *iro* system needed for the transport of FeGEnt.

**Table 1:** Siderophore nutrition and antibiotic susceptibility tests.

Siderophore	BN1071 ++ Fiu^+^, FepA^+^, Cir^+^	OKN359 *Δfiu, ΔfepA, Δcir* Fiu^−^, FepA^−^, Cir^−^	OKN9 *Δfiu* Fiu^−^, FepA^+^, Cir^+^	OKN35 *ΔfepA, Δcir* Fiu^+^, FepA^−^, Cir^−^	OKN359/pFiu Fiu^+^, FepA^−^, Cir-	OKN359/pFepA Fiu^−^, FepA^+^, Cir-	OKN359/pCir Fiu^−^, FepA^−^, Cir^+^
FeEnt	27 ± 0.5	0	27 ± 1.3	15 ± 0.7	14 ± 0.5	26 ± 2	11 ± 0.5
FeEnt*	23 ± 0.9	0	23 ± 0.8	24 ± 2	25 ± 2.5	24 ± 0.9	21 ± 0.2
FeGEnt	8.5 ± 0.1	8.5 ± 0.1	8.5 ± 0.1	8.5 ± 0.1	8.5 ± 0.1	8.5 ± 0.1	12 ± 0.1
FeDHBA	18 ± 0.1	0	18 ± 0.1	0	0	18 ± 0.1	12 ± 0.1
FeDHBS	0	0	0	0	0	0	12 ± 0.1
FeFDC	37 ± 2	0	32 ± 3	32 ± 2	30 ± 0.7	0	31 ± 3
FeMB-1	0	0	0	0	0	0	0

The values are diameters of growth or killing halos (measured with a ruler) around paper disks containing 100 μM of catecholate ferric siderophores or 50 μM siderophore antibiotics after incubation at 37°C for 24 h.

Bacteria expressing Fiu or Cir were highly susceptible to the iron complex of cefiderocol (FDC; Figure 1), which only requires OM transport to achieve bacteriocidal activity in the periplasm. Ferric siderophores, on the other hand, require both OM and IM transport for growth stimulation. Therefore, aside from revealing OM transport, the large FeFDC killing zones are not relatable to the sizes of siderophore nutrition test halos.

### Radioisotopic measurements of ferric siderophore uptake

**Figure 2:**
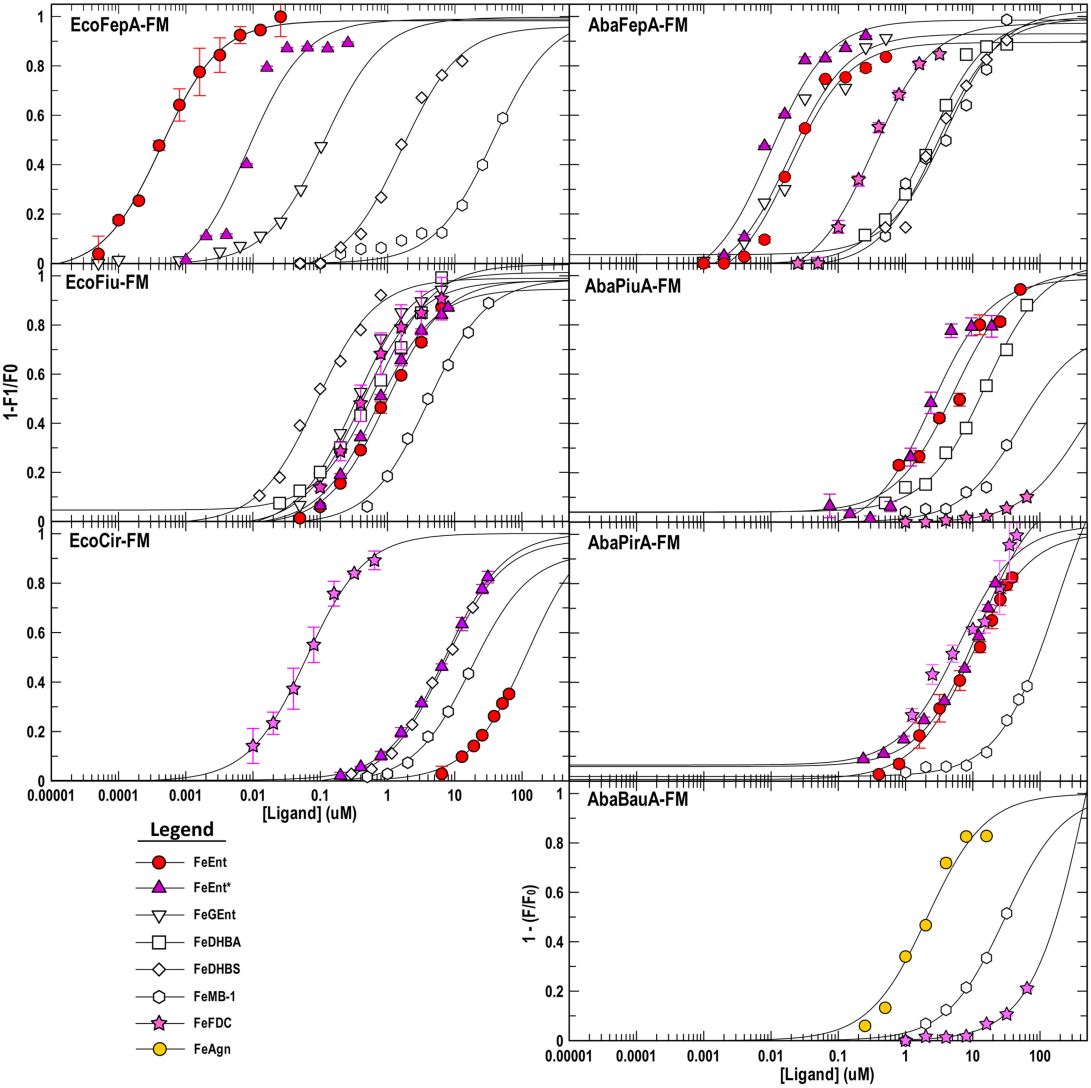
Ferric catecholate receptor binding titrations. We determined binding affinities (see Supplementary Figure S2), by titrating OKN1359 harboring plasmids expressing the noted Cys mutant receptors with ferric catecholates and plotting the concentration dependence of the ensuing fluorescence quenching. For each trial, after measuring each data point in triplicate, we calculated the mean fluorescence quenching and its standard deviation and then plotted 1−F/F_0_ versus ligand concentration. Data points represent the mean of the triplicate values, with associated standard deviations for trials with FeEnt, FeEnt*, and FeFDC. We fit the data to the 1-site with a background binding model of GraFit 6.0.12 that yielded the titration curves and resulting K_D_ values (see Table 2). The data depict a hierarchy of affinities among the different receptors for tri-, di-, and monocatecholate iron complexes. Relative to the prototypic high-affinity receptors EcoFepA and AbaFepA (Kumar et al., 2022), Fiu, Cir, and their orthologs/paralogs in *A. baumannii* show much lower affinity, focused on ferric di- and monocatecholates. The figure also illustrates the ability of the sensor library to monitor affinities over a 7-log range of concentrations, from sub-nanomolar to millimolar concentrations.

Radioisotopic iron accumulation measurements (Figure 1B) confirmed the preference of EcoFiu for FeEnt* that was observed in microbiological assays. The left panel in Figure 1 shows the uptake of [^59^Fe]Ent by FepA, a high affinity (K_D_ = 0.3 nM; Newton et al., 1999), specific process. Consequently, background uptake is negligible when we assayed FepA-deficient cells with nanomolar levels of FeEnt. The right panel, on the other hand, shows the uptake of [^59^Fe]Ent*, a lower affinity (K_D_ = 0.74 μM; Figure 2) process that is accomplished by Fiu, FepA, or Cir (Figures 1A, 2). The background of FeEnt* uptake by OKN359 (*Δfiu, ΔfepA, Δcir*) was higher, suggesting either non-specific adsorption of FeEnt*, or the presence of another, as yet unidentified uptake pathway. Nevertheless, bacteria expressing Fiu accumulated FeEnt* to levels that were 2–3 times higher than the background, confirming its ability to transport the degradation product. In summary, the wild-type parental strain BN1071, which does not produce any siderophores (*entA*) but expresses Fiu, FepA, and Cir, accumulated both [^59^Fe]Ent and [^59^Fe]Ent*, whereas two strains that expressed Fiu as their sole catecholate-specific LGP, OKN35 and OKN359/pEcoFiu (*fiu^+^*), acquired only [^59^Fe]Ent* but not [^59^Fe]Ent. These data reiterated the siderophore nutrition test results that EcoFiu transported FeEnt* but not FeEnt.

### Fluorescence spectroscopic measurements of receptor-ligand binding

To monitor the adsorption of ligands by EcoFiu, we engineered single Cys substitutions in its surface loops and alkylated them with FM (Supplementary Figure S2). When LGPs bind iron complexes, attached fluorophores may undergo concentration-dependent quenching, from direct contact between the ligand and the fluorophore, or conformational changes that relocate the fluor, energy transfer, or other mechanisms (Payne et al., 1997). Plots of fluorescence intensity versus [ligand] reveal binding affinity. Based on the Fiu crystal structure (Grinter and Lithgow, 2019a), we introduced substitutions S347C, T414C, S555C, and A694C in EcoFiu (Supplementary Figure S2). All the Cys mutants were active in siderophore nutrition tests with FeEnt* (data not shown). When we assessed the fluoresceination of the mutants in living bacterial cells, Fiu_A694C-FM showed the best labeling, so we used it to monitor the binding of different ligands. Titrations of OKN1359/pFiu_A694C-FM with increasing amounts of FeEnt, FeEnt*, FeGEnt, FeDHBA, FeDHBS, FeFDC, or FeMB-1 yielded K_D_ values for the binding interactions (Figure 2; Table 2). This fluorescent sensor methodology may also be used for rapid, stopped-flow measurements that resolve the different binding stages (Smallwood et al., 2014). However, the affinity determinations that we report herein yielded the overall affinity of the binding reaction at equilibrium, in the absence of transport (i.e., in TonB-deficient host strains).

**Table 2:** *E. coli* and *A. baumannii* ferric catecholate receptor affinities (ηM^1^ or μM).

	Siderophore							
Sensor	FeEnt	FeEnt*	FeGEnt	FeDHBA	FeDHBS	FeFDC	FeMB-1	FeAgn
EcoFepA	0.4 ± 0.04^1^	36 ± 9^1^	0.1 ± 0.04	NB	1.6 ± 0.3	NB	37 ± 11	NB
EcoFiu	1 ± 0.1	0.7 ± 0.07	0.34 ± 0.04	0.7 ± 0.1	90 ± 0.2^1^	0.40 ± 0.5	4.1 ± 0.5	NB
EcoCir	121 ± 31	7.3 ± 0.4	NB	NB	7.2 ± 0.9	63 ± 6^1^	18 ± 5	NB
AbaFepA	20 ± 5^1^	10 ± 3^1^	19 ± 5^1^	2.3 ± 0.4	0.3 ± 0.7	0.3 ± 0.07	3.9 ± 1.4	NB
AbaPiuA	5.3 ± 1.5	2.5 ± 0.9	NB	15 ± 3	NB	388 ± 247	51 ± 15	NB
AbaPirA	9.5 ± 1.5	12.5 ± 4	NB	9.4 ± 0.9	161 ± 40	6.2 ± 2.3	178 ± 61	NB
AbaBauA	NB	NB	NB	NB	NB	642 ± 451	31 ± 6	2.1 ± 0.4

The binding titrations in Figure 2 were fitted to a 1-site with background binding model using GraFit 6.012, that yielded the tabulated apparent K_D_ values and the associated standard errors of the fitted curves. NB, no binding. A preliminary report of some of these affinities appeared in Kumar et al. (2022).

^1^ηM K_D_ value.

### *Escherichia coli* ferric catecholate receptors

Fiu_A694C-FM differentiated ferric catecholate siderophores with the following order of affinities: FeDHBS (K_D_ = 0.1 μM) > FeGEnt (K_D_ = 0.34 μM) > FeFDC (K_D_ = 0.4 μM) > FeEnt* (K_D_ = 0.74 μM) > FeEnt (K_D_ = 1 μM) > FeMB-1 (K_D_ = 4 μM). We did not observe significant quenching at site A694C-FM from the addition of FeDHBA, but Fiu_N554C-FM, which is closer to the binding pocket (as computed by our docking study; see below), was quenched by FeDHBA (K_D_ = 0.7 μM). The defining aspect of Fiu’s binding activity was its ~1,000-fold lower affinity (micromolar K_D_ values) for any of the ferric catecholates, relative to the high affinity of EcoFepA (nanomolar K_D_ values) for FeEnt, FeEnt*, and FeGEnt (Figure 2).

### *Acinetobacter baumannii* ferric catecholate receptors

The susceptibility of *A. baumannii* to FeFDC (Zhanel et al., 2019; Delgado-Valverde et al., 2020; McCreary et al., 2021; Le et al., 2022) led us to investigate the recognition preferences of PiuA, AbaFepA, PirA, and BauA. The fluorescence spectroscopic binding determinations showed that the ligand preferences and affinities of these receptors for various ferric catecholate iron complexes were generally similar to those of their *E. coli* counterparts, with some exceptions.

### AbaFepA

The first difference was that AbaFepA did not show the same high affinity for its ligand(s) that defines EcoFepA (48% sequence identity) and its close orthologs [i.e., KpnFepA and KpnIroN (Kumar et al., 2022)]. The ~10 nM K_D_ value of AbaFepA for FeEnt indicated ~100-fold lower avidity for FeEnt than that of EcoFepA (0.1–0.3 nM; Newton et al., 1999; Chakravorty et al., 2019). Additionally, AbaFepA bound both FeEnt and FeEnt* with roughly the same affinity, whereas Eco FepA showed a strong preference (~100-fold) for FeEnt.

### PiuA

As noted above, Fiu bound FeEnt and FeEnt* with micromolar affinities that were 100- to 1,000-fold weaker than the nanomolar affinity of EcoFepA for FeEnt. The *A. baumannii* ortholog of Fiu, PiuA (31% sequence identity), similarly adsorbed FeEnt and FeEnt* with lower (~1,000-fold) affinity than AbaFepA. Furthermore, overall, the affinity of PiuA for FeEnt and FeEnt* was ~10-fold lower than that of Fiu.

### PirA

Although they are 37% identical, the ligand selectivities of Cir and PirA differed in a few ways. The former preferentially bound FeEnt* and FeDHBS better than FeEnt, whereas the latter had comparable affinities for FeEnt, FeEnt*, FeDHBA, and FeFDC. Cir did not adsorb FeDHBA. Its most distinguishing attribute was its comparatively high affinity (K_D_ = 60 nM) for FeFDC (Figure 2; Table 2).

### Recognition of FeFDC

Among the *E. coli* receptors, Cir and Fiu showed the best recognition of FeFDC (K_D_ values of O.06 and 0.5 uM, respectively; Figure 2; Table 2). The orthologs of *A. baumannii* bound FeFDC with lower affinity: AbaFepA (K_D_ ~ 0.5 uM), PirA (K_D_ ~ 5 uM), PiuA, and BauA (K_D_ > 100 uM). Unexpectedly, both Cir and PirA preferentially recognized FeFDC and bound it more avidly than any other iron complex. BauA’s natural ligand is the mixed catecholate/hydroxamate ferric acinetobactin (FeAcn), but it still showed weak affinity for FeFDC and FeMB-1 (Figure 2; Table 2). In summary, the fluorescent binding titrations revealed both similarities and differences in the hierarchies of ferric catecholate recognition by the LGP of *E. coli* and *A. baumannii* that were consistent with the susceptibility of both organisms to FDC.

### *In silico* predictions of ligand binding sites and molecular dynamics (MD) simulations

**Figure 3:**
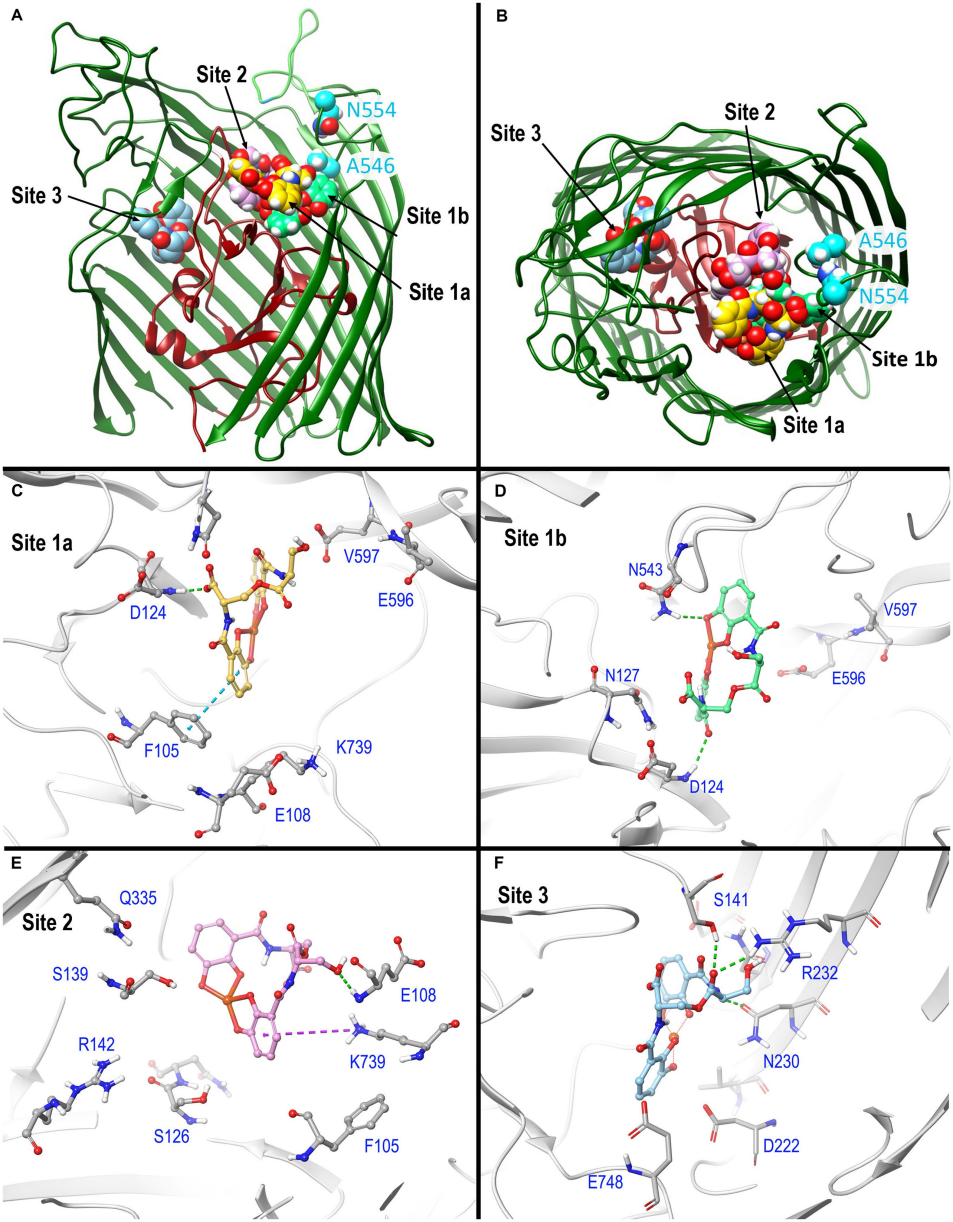
*In silico* docking/MD binding simulation. **(A)** AutoDock Vina Tool 1.5.6. paired Fiu (PDB 6BPM) and FeEnt*, to identify three outer binding sites (1a, 1b, and 2), and a fourth (site 3) deeper in the interior, within which the ferric siderophore is colored gold, spring green, orchid, and sky blue, respectively. We used the resulting docked model as the starting point for MD simulations that assessed the participation of individual amino acids in sites 1 and 2 during ligand recognition. In **(A)**, the terminal 11 β-strands of the barrel were rendered transparent to reveal the ligand’s positions. (B) A 90 degree rotation on the x-axis shows a surface view of the receptor, and the binding positions of FeEnt*. The first N-domain (dark red) loop (NL1) bifurcates the interior cavity. We fluoresceinated the engineered residues N554C (cyan) near site 1a and A546C (cyan) near site 2 to study the binding reaction. **(C–F)** Detailed interactions of FeEnt* in sites 1a, 1b, 2, and 3, respectively (see text for detailed explanations).

Using AutoDock Vina (Trott and Olson, 2010; Eberhardt et al., 2021), we identified potential FeEnt* binding sites in *E. coli* Fiu (PDB 6BPM). This initial analysis found three binding sites: two surrounded by surface loops 6, 7, 8, and 9, and a third site deeper in the Fiu structure (Figure 3), in the interior of the β-barrel. Site 1 was situated in a vestibule cavity (Grinter and Lithgow, 2019a) bounded by the N-domain loop and external loops 7, 8, and 9. Site 2, located more centrally in the vestibule, adjoins the N-domain loop, beneath external loop 6. Site 3 sits beneath L3 and atop β-strands 5 and 6. AutoDock Vina identified the top 9 binding poses in each of the three sites, and for each site we chose the one with the highest score for further analysis (Figure 3). Another AutoDock Vina simulation using Fiu and FeDHBA (that Fiu does not transport) found two binding sites within Fiu that involved different residues and different ligand binding configurations from those that we identified (see Discussion section). The large internal cavity in Fiu, which houses site 3, was also apparent in its structural model (Grinter and Lithgow, 2019a).

After the initial identification of binding sites by AutoDock Vina, we used its resulting docked model as the starting point for MD simulations that assessed the participation of individual amino acids in sites 1 and 2 during ligand recognition. Over a duration of 500 ns, the binding interactions in site 1 were dynamic and variable, encompassing numerous positions of the iron complex, that bifurcated site 1 into sites 1a and 1b (see below) (Figure 3A). FeEnt* initially entered site 1a, near the conjunction of the first N-domain loop (NL1) and surface loop 9 (L9) of the β-barrel. The model predicted that in Site 1a, the negatively charged (−2), aromatic iron center of FeEnt* interacted with F105 (π-π stacking), D124 (H-bond), and K739 (ionic and cation-π bonds). In site 1a, FeEnt*was in proximity to NL1 (residues 119–123), L8 (residues 593–596), and L9 (residues 655–658) (Figure 3B). The siderophore’s four catecholate oxygens associated with Fe^3+^, along with two oxygens from extrinsic water molecules, creating an overall hexacoordinate metal complex (Figure 3).

After initial adsorption in site 1a, FeEnt* moved to site 1b, a binding pocket formed by residues 123–127 (NL1), 165–167 (NL2), and 538–544 (L7), wherein it hydrogen bonded to D124, N127, and N543. FeEnt* next occupied site 2, adjacent to and slightly beneath site 1, near the conjunction of the N-domain loop and L6. In site 2, the binding contacts were more stable. The simulations predicted interactions with F105 (π-π stacking), S126, N127, S139 (H-bonds), and K739 (cation-π interaction). The basic side chain of R142 was highly dynamic and only 4.3–9 Å away from the catecholate siderophore during the 500 ns simulation experiment. R142 may form a cation-π bond with the electron-rich aromatic ring, an ionic bond, or both, as the negatively charged ferric siderophore moves deeper in the beta-barrel. Residues 106–107 and 111–113 in NL1 were also in close contact with the ligand. When FeEnt* occupied binding site 2, it H-bonded to the amide nitrogen of E108, whose γ-carboxyl neared ionic bond distance to R486 in L6, that closed over the bound ligand (see below). Together with D124, E108 may play a key role in the translocalization of ligands from site 1a to site 1b and finally to site 2.

The movement of ligand from 1a to 1b occurred through two transition states (Supplementary Figure S4). Site 1a is ~10 Å removed from site 1b; the translocalization of FeEnt* between these sites is perhaps more accurately described as an induced fit of Fiu to capture the ferric siderophore from the environment.

### Open and closed conformations of Fiu

**Figure 4:**
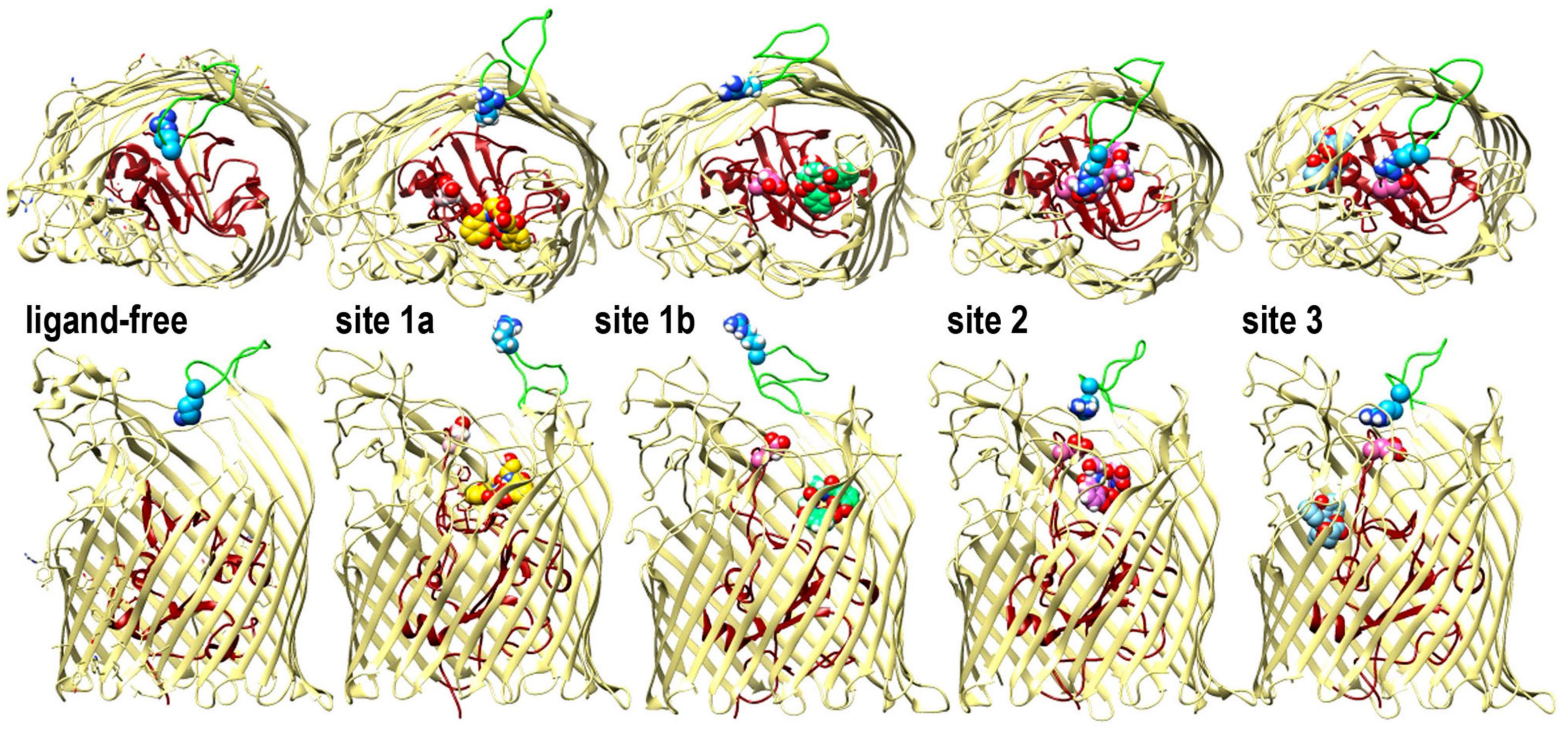
Open and closed conformations of Fiu. The images depict the predictions of MD simulations over 500 *n*s during FeEnt* binding to Fiu, as ribbon diagrams of Fiu either ligand-free, or associated with FeEnt* in sites 1a, 1b, 2, or 3. FeEnt* is colored gold, spring green, orchid, or sky blue, in sites 1a, 1b, 2, or 3, respectively. Surface loop conformations during the binding reaction are shown from above (top) or from the side (below); the N-domain is colored dark red. Although β-strands and other loops (khaki) remained relatively stationary, L6 (green loop) fluctuated among open conformations during initial FeEnt* binding to site 1a, but adopted a closed conformation above FeEnt* when the iron complex moved to site 1b. In the side view we rendered loops 3 and 10 transparent to better reveal sites 1a and 1b, see also Supplementary Video S1.

In addition to the predictions of FeEnt* binding sites, the MD simulations showed several configurations of surface loop 6 during the binding reaction (Figure 4). L6 was conspicuously open in sites 1a and 1b, but as FeEnt* progressed to site 2, L6 closed over the metal complex, with residue R486 dynamically moving toward N111, N544, or bound FeEnt*. The interactions between R486 in L6 and E108 in NL1 were particularly noteworthy because in site 2 the guanidino moiety of the R486 side chain approached the carboxylate of E108 to a distance of 5–6 Å, near salt bridge proximity. Grinter and Lithgow (2019a) reported two conformations of Fiu in the absence of a ligand that involved different forms of the N-domain loop. The changing surface loop configurations that we observed during the simulated Fiu-FeEnt* binding reaction were different, in that they encompassed the motion of L6 during ligand binding. These MD results are the first indication of loop motion in Fiu to enclose FeEnt*. However, in this respect, Fiu mimics the induced fit binding mechanisms of FepA (Scott et al., 2002; Smallwood et al., 2014) and FecA (Ferguson et al., 2002) as they close around their iron complexes. Hence, dynamic motion to capture ligands by induced fit may be a general property of LGP binding reactions.

### Biochemical analysis of predicted binding sites in Fiu

Using *fiu^+^* in the low-copy vector pITS23, we introduced Cys substitutions in L7 for fluoresceination, near binding sites 1a and 1b (FiuA546C) and 2 (FiuN554C). We then combined the Cys mutants with single or double Ala substitutions for residues of interest in sites 1 and 2. Our biochemical analyzes focused on sites 1a and 2 because site 3, deeper in the interior, was too distant from the L7 surface-localized fluorophores to permit analysis of its component residues by this approach. After confirmation of the mutations in sites 1 and 2 by DNA sequencing, we transformed the plasmids into *E. coli* OKN359 for evaluation in siderophore nutrition tests and into OKN1359 (*ΔtonB, Δfiu, ΔfepA, Δcir*) for fluorescent labeling and binding affinity determinations. We grew the resulting strains in iron-deficient MOPS media and subjected their engineered Cys side chains to chemical modification with FM. Fluorescence image analysis of SDS-PAGE gels of OM fractions from the mutants showed that all were expressed at or near wild-type levels and were well-labeled by FM (Supplementary Figure S3). The concentration-dependent fluorescence quenching of each construct by FeEnt* defined the binding affinities of the Fiu mutants. The experiments first showed that the Cys mutations near the EcoFiu binding pockets did not hinder the adsorption of FeEnt* (Table 3). Next, the individual Ala substitutions in binding site 1a behaved much like wild-type Fiu: They had slightly (~2-fold) lower affinity for FeEnt* than the positive control, N554C (K_D_ = 0. 9 μM): F105A (K_D_ = 1.2 μM); E108A (K_D_ = 1.7 μM); D124A (K_D_ = 2.5 μM); K739A (K_D_ = 1.8 μM). The single mutants in binding site 2, however, were more impaired relative to the positive control A546C (K_D_ = 1 μM). R142A showed the lowest affinity of FeEnt* binding (K_D_ = 2.7 μM) of any single mutant. Other mutations in binding site 2 had smaller effects: S126A (K_D_ = 1.9 μM), N127A (K_D_ = 1.6 μM), S139A (K_D_ = 1.7 μM), and Q335A (K_D_ = 1.9 μM).

**Table 3:** Binding and uptake of FeEnt* by Fiu and its Cys or Ala substitution mutants.

Mutation	fiu^+^	Cys mutants		Single Ala mutants: Site 1				Single Ala mutants: Site 2					Double Ala mutants			
		N554	S546	F105	E108	D124	K739	S126	N127	S139	R142	Q335	F105-R142	E108-R142	D124-R142	K739-R142
Affinity^a^	ND	0.9 ± 0.2	1.0 ± 0.1	1.2 ± 0.1	1.7 ± 0.2	2.5 ± 0.1	2 ± 0.1	1.9 ± 0.1	1.6 ± 0.1	1.7 ± 0.1	2.7 ± 0.1	2 ± 0.1	8 ± 1	6 ± 0.1	9 ± 0.6	19 ± 2
Uptake^b^	28 ± 0.1	27 ± 1.4	26 ± 0.5	16 ± 0.7	0	0	0	26 ± 0.7	27 ± 0.7	26 ± 0.7	8 ± 0.1	25 ± 0.7	0	0	0	0
Nutrition Tests^c^	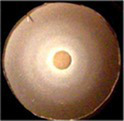	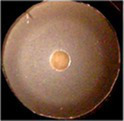	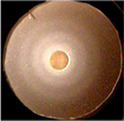	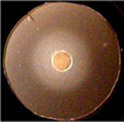	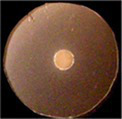	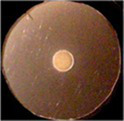	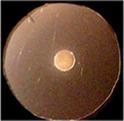	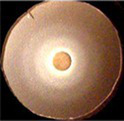	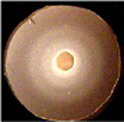	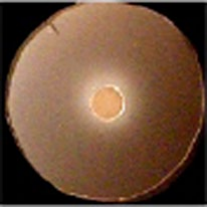	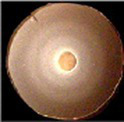	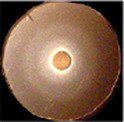	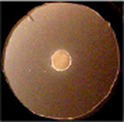	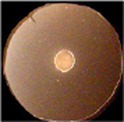	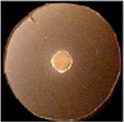	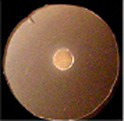

a Apparent K_D_ values (μM) for the binding reaction with FeEnt*, determined by quenching titrations of fluorescently labeled, genetically engineered. Cys sulfhydryl near predicted binding sites. ND: no data for wild-type Fiu that were not labeled by FM.

b Diameters (mm) of growth halos in siderophore nutrition tests with 10 μL of 100 μM FeEnt* applied to a paper disk on NB agar containing 100 μM apoferrichrome A, after incubation at 37°C for 24 h.

c OKN359 harboring pHSG575 derivatives carrying *fiu*^+^or its mutant alleles was grown in NB and plated on NB plates. 10 uL of 100 μM FeEnt* were applied to a paper disk on the agar, and the plates were incubated overnight at 37°C.

**Figure 5:**
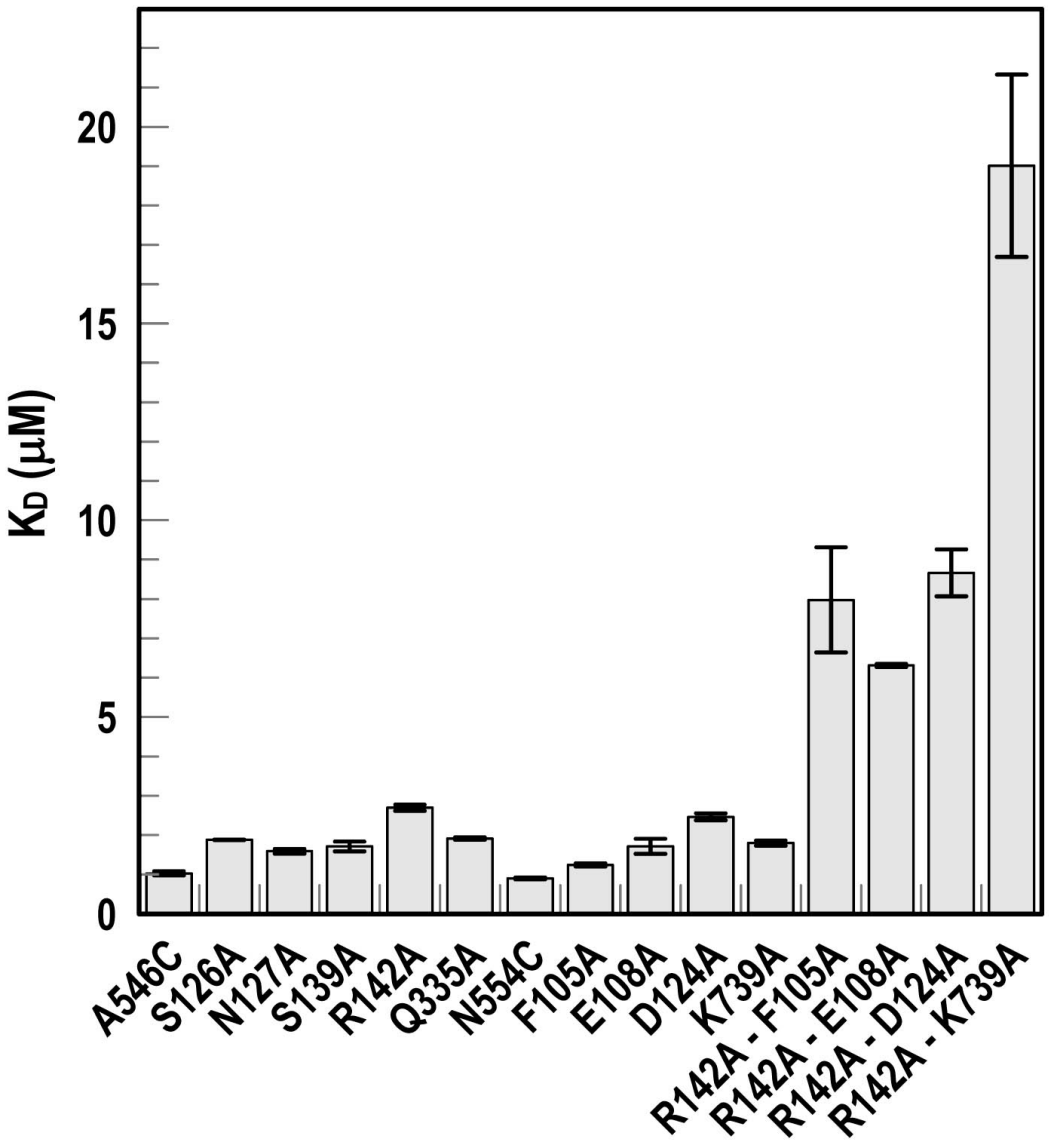
Ligand binding by Ala substitution mutants. In predicted binding sites 1a and 2, we engineered sensor residues A546C and N554C, respectively, and then engineered Ala substitutions for 9 other residues in the two binding cavities, as well as double mutation combinations. After the expression of the Fiu Cys-Ala mutants in OKN1359, we fluoresceinated the cells and determined their affinities for FeEnt* by fluorescence quenching.

Although single Ala substitutions in a multi-determinant binding pocket may only show small impacts on functionality, double substitutions for participant side chains often create larger reductions in ligand binding (Newton et al., 1997). Hence, we generated several double Ala substitutions, that combined single mutations in site 1a (F105A, E108A, D124A, K739A) with R142A, which created the most noticeable effects in site 2. After verifying the correct expression and fluorescence labeling of the double mutants (Supplementary Figure S3), we measured their binding affinities by fluorescence quenching assays. All four constructs had impaired binding relative to the positive controls and the single mutants, with a 7- to 22-fold increase in K_D_. The greatest decrease in affinity (22-fold) was from K739A-R142A (Figure 5; Table 3).

### Effects of Ala substitutions on FeEnt* uptake

In addition to binding affinity titrations, we assessed the transport efficiencies of the Ala substitution mutants with siderophore nutrition tests. The experiments further illustrated the contributions of residues in sites 1a and 2. For example, despite the fact that none of the four single substitutions in site 1a substantially changed binding affinity (≤ 2-fold), three of those single mutants (E108A, D124A, and K739A) abolished the transport of FeEnt*. Even at 100 μM, FeEnt* did not produce growth halos on the lawns of these mutants. Similarly, F105A, also in site 1a, showed weaker growth than the wild-type control. The outcomes were similar but different in site 2. As in site 1 the single Ala substitutions only slightly decreased binding affinity (2- to 3-fold), and most had little impact on FeEnt* uptake: neither S126A, N127A, S139A, nor Q335A reduced growth on FeEnt* relative to the positive control FiuN554C. Only R142A in site 2 severely diminished the halo around FeEnt*. Overall, these data showed that changes in either predicted site could disrupt FeEnt* transport. Analysis of the double Ala substitution mutants supported this interpretation because all of the four site 1a–site 2 double mutants were completely defective in FeEnt* uptake. Thus, Ala substitutions in either site 1a or site 2 affected FeEnt* recognition, binding, and uptake.

## Discussion

### Acquisition of ferric catecholates

The findings clarify the transport attributes of *E. coli* Fiu, that was initially thought to be responsible for the uptake of FeDHBS, a monocatecholate degradation product of FeEnt (Hantke, 1990). Our results indicate that although Fiu adsorbs FeDHBA, FeDHBS, FeFDC, and FeMB-1, ferric monocatecholates are not its primary transport target. Rather, Fiu preferentially transported the dicatecholate degradation product, FeEnt*. Among numerous ferric catecholates that we tested, Fiu only transported FeEnt* and the siderophore antibiotic FeFDC. Cir had identical ligand specificity for FeEnt* and FeFDC. Aside from the initial reports of its uptake of FeDHBS (Hantke, 1990) and catecholate-β-lactam antibiotics (Nikaido and Rosenberg, 1990), the transport specificities of Cir were largely unexplored. Our data confirm its transport of FeDHBS and monocatecholate antibiotics (FeFDC); our findings additionally demonstrate, for the first time, its recognition and transport of FeEnt*. Despite its highest affinity and specificity for FeEnt, FepA also bound and transported both FeEnt* and FeDHBA, further illustrating the importance of these degradation products to overall iron acquisition. However, cells expressing only FepA were not susceptible to the monocatecholate FeFDC. These preferences of the three chromosomal ferric catecholate receptors of *E. coli* strategically balance to accommodate the tendency of FeEnt to decompose/degrade from the susceptibility of its lactone backbone to acid or base, or cellular hydrolases that cleave the lactone ring. The latter process sequentially produces linear trimeric and then dimeric ferric catecholate species that FepA, Fiu, and Cir recognize and transport. FepA is most important in the overall cell envelope transport system, because its higher affinity for both FeEnt and FeEnt* allows their uptake at even nanomolar concentrations. Fiu and Cir, on the other hand, add additional iron transport capability when ferric catecholates are present at much higher, micromolar levels.

Regarding the ferric catecholate recognition specificities of *A. baumannii,* relative to *E. coli,* genomic annotations from sequence identities among the various receptors generally agreed with their biochemical activities. That is, AbaFepA was most like EcoFepA, in sequence identity and its specificity for FeEnt, etc., with one exception: PirA was more identical to EcoFepA (56%) and AbaFepA (53%) than to Cir (36%), its purported ortholog (Supplementary Figure S6). This higher level of sequence identity between PirA and EcoFepA or AbaFepA inferred that its true ligand is FeEnt. However, the fluorescent binding titrations did not support this prediction: PirA showed no selectivity for FeEnt, but instead, similar micromolar affinity for FeEnt, FeEnt*, FeDHBA, and FeDHBS. Its affinity for FeEnt was 10^5^- and 10^3^-fold lower than that of EcoFepA and AbaFepA, respectively.

### Biological relevance of Fiu, Cir, and FepA specificities

**Figure 6:**
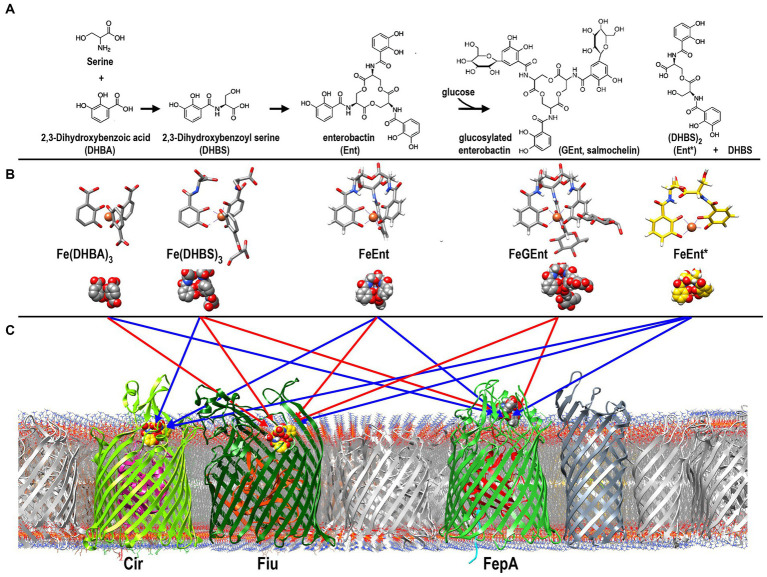
Pathways of ferric catecholate binding and transport in *E. coli*. **(A)**. Catecholate siderophore biosynthesis and degradation. Ent is a trimer of DHBS, whose serine hydroxyl and α-carboxylate groups esterify to form a trilactone ring; the α-amino of Ser forms an amide bond with the carboxylate of DHBA. The resulting trimer may be glucosylated to form GEnt; both Ent and GEnt, and their iron complexes, may be degraded by acid, base, or esterases to form FeEnt* and DHBS. **(B)** Ferric complexes of catecholate siderophores. The monocatecholate biosynthetic and degradative byproducts, DHBA and DHBS, form relatively low affinity hexacoordinate, octahedral complexes with Fe^3+^. The tri- and dicatecholates Ent, Gent, and Ent*, on the other hand, form a higher affinity, hexacoordinate iron chelates. **(C)** Binding and uptake of ferric catecholates by Fiu, Cir, and FepA. The findings that we report established pathways of ferric catecholate binding only (red arrows), and binding followed by transport (blue arrows). In summary, Fiu only transported FeEnt*, but it also bound FeDHBA, FeDHBS, FeEnt, and FeGEnt; Cir transported FeEnt* and FeDHBS (weakly), but it also bound FeEnt; FepA transported FeDHBA, FeEnt, and FeEnt*, but it also bound FeGEnt, albeit with a much lower affinity.

In response to iron deprivation, Fiu, FepA, and Cir are over-expressed to a level that is 10- to 20-fold higher than in iron-replete media, and the kinetics of their de-repression are virtually identical (Klebba et al., 1982). This coordinate, Fur-regulated enhancement of the three ferric catecholate transporters accompanies and mimics the Fur-regulated overproduction of Ent that also ensues from iron deprivation. The synchronous regulation of the Ent biosynthetic and the FeEnt/FeEnt* uptake systems concurs with our finding that all three receptors participate in the acquisition of FeEnt or its degradation products. However, despite their similar patterns of overproduction during low-iron stress, the different affinities of the three ferric catecholate receptors dictate their transport physiology: Only FepA (K_D_^FeEnt^ = 0.4 nM) acquires FeEnt at low (nanomolar – micromolar) concentrations, but all three proteins may transport FeEnt* (FepA: K_D_ = 40 nM; Fiu: K_D_ = 0.7 uM; Cir: K_D_ = 7 uM), which may accumulate at much higher (mM) concentrations during prolonged growth in iron-deficient conditions (Kumar et al., 2022) (Figure 6).

### Ligand recognition and movement of FeEnt* through Fiu

Despite its wide range of ferric catecholate recognition, Fiu only transported FeEnt*. The uptake of FeEnt*, but exclusion of FeEnt and Fe(DHBS)_3_, may likely derive from the larger size of the latter two iron complexes (555 vs. 719 and 774 Da, respectively). All three compounds manifest hydrophobicity from their peripheral aromatic rings and a negatively charged iron center (^−^2, ^−^3, ^−^3, respectively); the obvious difference among them is their overall size: FeEnt* is the smallest in mass and the most compact molecule. These considerations suggest that during uptake, FeEnt* travels a pathway that neither FeEnt nor Fe(DHBS)_3_ can follow, probably because of their larger size.

The identification of FeEnt* as Fiu’s natural ligand allowed AutoDock Vina to predict its binding interactions. The resulting docking model with FeEnt*, and ensuing MD simulations, showed binding positions in the Fiu vestibule and interior that delineated a putative pathway of ligand movement. These predictions had experimental support from the fact that site-directed Ala mutants in sites 1 and 2 impaired the binding and uptake of FeEnt*. The MD calculations further postulated the closing of L6 over FeEnt* when it moved to site 2 in Fiu, which mimics the two-stage kinetic model of FeEnt binding to FepA (Payne et al., 1997), and the closing of L7 in FecA during ferric citrate binding (Ferguson et al., 2002). Furthermore, when L6 changed position to cover FeEnt* in site 2, it was potentially stabilized by an ionic bond between R486 in L6 and E108 in NL1 (Figure 4). A similar phenomenon was observed in the FecA-ferric dicitrate crystal structure, as a salt bridge between residues R438 and E573 that covered the bound metal complex (Ferguson et al., 2002). In most circumstances, Fiu residues R142 and K739 have positively charged side chains that may form strong cation-π interactions with the ligand. This indicates the importance of amino acids with positively charged side chains and the associated cation-π bond in the bio-molecular complexing.

### TonB-dependent transport

The process of metal transit through TonB-dependent LGP is not fully understood, but accumulating information, including current and prior findings about Fiu (Grinter and Lithgow, 2019a), suggests a mechanism. Conformational motion within the globular N-domain of EcoFepA is one aspect of the FeEnt uptake mechanism (Majumdar et al., 2020). As noted above, Fiu transported FeEnt* but not larger ferric catecholates that bound with about the same affinity. The AutoDock Vina and MD simulations projected a series of binding sites in the protein interior that ultimately position FeEnt* above a narrow opening between the N-domain and the β-barrel (Supplementary Video S1). In its initial state (Grinter and Lithgow, 2019a), this pore is too small to permit passage of FeEnt*, implying that the role of TonB activity is to transiently enlarge the channel, either allowing or compelling FeEnt* into the periplasm (Supplementary Video S1). In this context, the pathway enlarges enough to accommodate the transit of FeEnt* but not of FeEnt nor Fe(DHBS)_3_.

Most mechanistic studies of LGP-mediated ferric siderophore transport involved *E. coli* FepA (Newton et al., 1999; Scott et al., 2002; Cao et al., 2003; Chakraborty et al., 2003; Ma et al., 2007; Newton et al., 2010; Smallwood et al., 2014; Majumdar et al., 2020), FhuA (Carmel and Coulton, 1991; Braun et al., 1994; Bonhivers et al., 1996; Moeck et al., 1997; Endriss et al., 2003; Faraldo-Gómez et al., 2003; Eisenhauer et al., 2005), or BtuB (Hunter and Glass, 1982; Gudmundsdottir et al., 1989; Bradbeer and Gudmundsdottir, 1990; Fanucci et al., 2002; Cadieux et al., 2003; Chimento et al., 2003; Shultis et al., 2006; Nyenhuis et al., 2020), but the crystal structure of Fiu (Grinter and Lithgow, 2019a) opened new avenues to understanding this process. The uptake of Trojan horse antibiotics such as FDC by Fiu (this report; Ito et al., 2018, 2019) adds medical relevance to its recognition and transport properties. Previous research on FepA provides some insight into LGP transport biochemistry. FepA adsorbs FeEnt in a biphasic binding reaction (Payne et al., 1997) that involves induced fit by its surface loops around the ferric siderophore (Jiang et al., 1997; Scott et al., 2002; Smallwood et al., 2014). After reaching binding equilibrium in the surface vestibule, interactions between the TonB C-terminus and the FepA N-terminus induce a conformational change in the receptor that results in the internalization of the iron complex into the periplasm (Bradbeer, 1993; Newton et al., 2010; Klebba, 2016). Consistent with this idea, the MD-predicted sequence of binding sites through the Fiu interior suggests FeEnt* movement and ultimate deposition in a site where small changes in N-domain conformation may allow ligand transfer into the periplasm. In this sense, the MD simulations also fit with data on ferrichrome passage through FhuA (Eisenhauer et al., 2005) and FeEnt uptake through FepA (Majumdar et al., 2020), that implied conformational change in the N-terminal globule, while resident in the β-barrel, as part of the ligand uptake mechanism.

Previous findings also frame the actions of TonB-ExbBD in the uptake reaction: (i) The TonB C-terminus physically engages the N-terminal TonB-box sequence of ligand-bound LGP at the OM - periplasm interface (Pawelek et al., 2006; Shultis et al., 2006); (ii) TonB undergoes rapid, PMF-dependent motion, probably rotation in the IM bilayer (Jordan et al., 2013); (iii) ionic bonds restrict the N-terminus within the β-barrel, as a result of basic side chains in the N-domain that pair with acidic side chains on the interior of LGP β-barrels (Klebba, 2003). In Fiu, salt bridges exist between R121 – E570 and K154 - E617. These basic–acidic side-chain pairs are 90–95% conserved and evolutionarily covariant at comparable positions in dozens of other LGP (Klebba et al., 2021). Furthermore, directly across the channel from the double salt bridges, a peptide linkage connects the N-domain to the β-barrel (in Fiu, at residue 181). Hence, ionic and peptide bonds constrain the N-terminus in the channel at two opposing positions, so force or torque imparted to the N-domain by TonB’s motion may cause conformational dynamics in the N-domain that enlarge the small pore, allowing FeEnt* to enter the periplasm and bind to FepB. This model suggests that PMF-driven TonB action drives conformational change that opens a precisely sized channel in the LGP interior, compelling ferric siderophore uptake (Supplementary Video S1).

FDC is licensed for the treatment of antibiotic-resistant Gram (−) bacteria, such as carbapenem-resistant *A. baumannii* and *P. aeruginosa* (Yamano, 2019; Shields, 2020; Wu et al., 2020). Our experiments identified *E. coli* Fiu and Cir as its OM uptake portals, which concur with reports of FDC uptake by Fiu and Cir orthologs in *A. baumannii* and *P. aeruginosa* (PiuA and PirA; Kim et al., 2015; Ito et al., 2018; Luscher et al., 2018). FDC has a monomeric molar mass of 752 Da; its tricatecholate iron complex is 2,306 Da, which makes it uptake through any LGP inexplicable. Additional experiments are needed to understand its transport. The understanding of EcoFiu and its orthologs will aid the further development of siderophore antibiotics (Lasko and Nicolau, 2020; Morris et al., 2020; Wu et al., 2020; Mabayoje et al., 2021) such as FDC. Given their overall structural (Ferguson et al., 1998; Locher et al., 1998; Buchanan et al., 1999; Ferguson et al., 2002; Chimento et al., 2003; Cobessi et al., 2005a,b; Buchanan et al., 2007; Cobessi et al., 2010; Grinter and Lithgow, 2019a,b; Grinter and Lithgow, 2020) and sequence (Klebba et al., 2021) commonalities, other LGPs likely internalize metal complexes, including Trojan horse antibiotics, by the same or similar mechanisms.

## Materials and methods

### Bacterial strains and plasmids

We utilized derivatives of *E. coli* strain BN1071 (*entA, pro, trp, B1, rpsL*; Klebba et al., 1982) carrying site-directed chromosomal deletions (Ma et al., 2007): OKN1 (*ΔtonB*), OKN3 (*ΔfepA*), OKN5 (*Δcir*), OKN9 (*Δfiu*), OKN35 (*ΔfepA, Δcir*), OKN359 (*ΔfepA, Δcir, Δfiu*), and OKN1359 (*ΔtonB, ΔfepA, Δcir, Δfiu*). We also studied CP9, an extraintestinal pathogenic *E. coli* strain from a patient with sepsis (Russo et al., 1993; Johnson et al., 1997; Nazareth et al., 2007); courtesy of Dr. Thomas A. Russo, University of Buffalo School of Medicine. For the generation of site-directed substitution mutants in *fiu,* we PCR-amplified the structural gene from the prototypic wild-type *E. coli* strain MG1655 (Blattner et al., 1997) and cloned it in the low-copy plasmid pHSG575 (Takeshita et al., 1987), under the control of fur-regulated *fepA* promoter (Kumar et al., 2022). We transformed OKN1359 and OKN359 with the resulting construct (pITS42) for binding and transport experiments, respectively.

### Site-directed mutagenesis

We used QuickChange (Stratagene) to create site-directed substitutions in *fiu,* carried on pITS42. With a pair of complementary primers that flank the target codon, we introduced mutations that encoded Cys or Ala residues in mature Fiu. After digesting the wild-type template DNA with DpnI, we transformed BN1071 with the mutant plasmid, isolated transformant clones, and sequenced their plasmids to confirm the substitutions in *fiu.*

### Bacterial culture conditions

OKN359 or OKN1359 carrying pITS42, that encoded WT or mutant *fiu* alleles, was grown in Luria-Bertani (LB) broth at 37°C overnight to stationary phase. For host strain and plasmid selection, the media contained streptomycin (100 μg/mL) and chloramphenicol (20 μg/mL), respectively. To impose low-iron stress and induce the expression of fur-regulated *fiu*, we sub-cultured stationary phase LB cultures at 0.5% into MOPS iron-deficient minimal medium (Neidhardt et al., 1974) and shook the flasks at 200 rpm for 20 h at 37°C, to a cell density of 1.5–2.5 × 10^9^ cells/mL.

### Siderophore purification

After growing *E. coli* strain AN102 (Wayne et al., 1976) to late log phase in T-media (McIntosh and Earhart, 1976), we purified Ent and incubated an aliquot of the aposiderophore with equimolar ^56^FeCl_3_ or ^59^FeCl_3_ to form the ferric siderophore (Wayne et al., 1976; Newton et al., 2010). Passage of the mixture over Sephadex LH20 in 5 mM NaHPO_4_ separated FeEnt and FeEnt* (Wayne et al., 1976). We also purified catecholate ferric siderophores from the pathogenic *E. coli* strain CP9 (Russo et al., 2002, 2011, 2015) that yielded FeEnt, FeGEnt, and their degradation product, FeEnt*. After growth in LB broth to stationary phase, we sub-cultured CP9 at 1% into T-medium and shook the flasks at 200 rpm for 30 h at 37°C, to a cell density of ~1.5 × 10^9^ cells/mL. We removed the bacteria by centrifugation at 5000 x g for 20 min and added FeSO_4_ to 5 mM in the culture supernatant to form ferric siderophores. After overnight incubation, we adsorbed the negatively charged ferric catecholates to Whatman DE52 cellulose in 50 mM Tris-Cl, pH 7, washed the resin with 5 column volumes of the same buffer, and eluted the iron complexes with a gradient of 0–2 M ammonium chloride. After collecting and concentrating the three red, dark red, or purple fractions (FeEnt, FeEnt*, and FeGEnt, respectively) by rotary evaporation, we desalted them by chromatography on Sephadex G-10 in 5 mM NaHPO_4_ and individually purified them on Sephadex LH20 in 5 mM NaHPO_4_, pH 7. LH20 columns yielded three distinct peak fractions that mass spectrometry identified as FeGEnt, FeEnt*, and FeEnt, respectively (Supplementary Figure S1). We spectroscopically measured the concentrations of FeEnt, FeEnt*, and FeGEnt, using the extinction coefficient of the former: 5.6 at 495 nm. The extinction maxima of both FeEnt* and FeGEnt are slightly red-shifted (503 and 510 nm, respectively). The only possible ambiguity regarding concentration involved FeEnt*, whose extinction coefficient at 503 nm is not yet known. However, given its comparable iron complex to that of FeEnt, we expect an extinction coefficient that is similar in magnitude. We determined the concentrations of the monocatecholate ferric siderophores from the stoichiometry of forming their iron complexes.

### Siderophore nutrition tests

We qualitatively analyzed iron transport by microbiological nutrition tests (Wayne and Neilands, 1975). Cells expressing wild-type Fiu or mutant derivatives were grown overnight in LB broth to stationary phase and sub-inoculated at 1% into nutrient broth (NB) containing streptomycin (100 ug/mL) and chloramphenicol (20 ug/mL). After overnight growth in NB, we mixed 100 μL of NB culture with 3 mL of NB top agar containing 100 μM apoferrichrome A and appropriate antibiotics, and put the mixture in a 6-well microplate. We applied a paper disk to the surface of the solidified agar and added 5 μL of 100 μM ferric siderophore to the disk. After 24 h at 37°C, we measured the diameter of growth around the paper disk. Ferric monocatecholate complexes (which have a much lower affinity for Fe^3+^) may release iron to the non-utilizable iron chelator apoFcA. Consequently, we modified the nutrition tests of ferric monocatecholate complexes to incorporate the lower affinity, non-utilizable chelator bipyridyl (0.5 mM) to complex adventitious iron in the media (Newton et al., 2005), instead of apoFcA.

### Radioisotopic ferric siderophore uptake assays

After growth in iron-deficient MOPS minimal media containing appropriate antibiotics, with shaking at 200 rpm for 5.5 h. at 37°C, we sampled 3 mL aliquots of bacterial culture and added ^59^FeEnt or ^59^FeEnt* to a final concentration of 10 μM. At t = 0, 5, 10, 15, 30, and 45 min, we sampled 100 μL of bacterial culture from the flask, collected the cells on 0.45 um membrane filters, washed the filters with 10 mL of 0.9% LiCl, and determined amount of [^59^Fe] on each filter by counting in Packard gamma counter. For each time point, we collected triplicate samples and determined the mean CPM.

### Preparation of OM fractions

Following growth in iron-deficient MOPS minimal media, with shaking at 200 rpm for 20 h at 37°C, we harvested the bacterial cells by centrifugation at 5000 x *g* for 20 min and resuspended the pellet in 4 mL of PBS containing trace amounts of RNase and DNase. We lysed the cells by passage through a French pressure cell at 14,000 psi, removed debris and unbroken cells by centrifugation at 5000 x *g* for 20 min, and centrifuged the supernatant in a microfuge at 13000 rpm for 1 h to pellet the cell envelope fraction, that we resuspended in 200 μL of 50 mM Tris–HCl, pH 7.4. After adding an equal volume of 1% sodium sarcosinate, we incubated the mixture with gentle mixing for 30 min at room temperature to solubilize the IM fraction and then sedimented the OM by centrifugation at 13000 rpm for 1 h. We resuspended the OM pellet in 200 μL of 50 mM Tris–HCl, pH 7.4 and stored it frozen at −20°C.

### SDS-PAGE

We analyzed protein expression and fluorescence labeling by SDS-PAGE (Lugtenberg et al., 1975; Hancock and Braun, 1976). For determinations of protein expression, we solubilized 50 μg of the OM fraction (determined by absorbance at 280 nm) in a sample buffer containing 1% SDS and 0.3% β-mercaptoethanol, at 100°C for 5 min. We resolved the OM proteins on 12% acrylamide /0.3% bis-acrylamide SDS-PAGE gels (Ames, 1974; Lugtenberg et al., 1975; Hancock and Braun, 1976), observed fluorescently labeled Fiu Cys mutant proteins by transillumination with UV light, and then stained the gels with Coomassie blue to observe the Fiu expression relative to that of other LGPs. For experiments with FM-labeled bacterial cells, we mixed 10^8^ bacteria with SDS sample buffer, boiled the sample for 5 min, and performed SDS-PAGE as described above.

### Site-directed fluorescence labeling with FM

We cultured strains expressing Cys mutant Fiu derivatives in 10 mL of MOPS iron-deficient minimal media for ~20 h, to a density of 2–3 × 10^9^ cells/mL (Newton and Klebba, 2022). After collecting the bacteria by centrifugation at 7000 x *g* for 10 min, we washed the pellet with and resuspended it in 10 mL of 50 mM NaHPO_4_ pH 6.7. We added FM to the cell suspension to 5 μM, incubated the mixture for 15 min 37°C, and quenched labeling by adding β-mercaptoethanol to 100 μM. The fluoresceinated bacterial cells were pelleted by centrifugation at 7000 x g for 10 min, washed with and resuspended in 10 mL of PBS pH 7.4, followed by SDS-PAGE/image analysis of labeling specificity, and spectroscopic determinations of fluorescence intensity.

### Fluorescence determinations of ligand binding

We performed fluorescent determinations of ligand binding in an SLM AMINCO 8100 fluorescence spectrometer with an OLIS operating system (OLIS Inc., Bogart, GA). We added 10^8^ FM-labeled bacterial cells to 2 mL of PBS in a quartz cuvette, with stirring. Excitation and emission wavelengths were 488 nm and 520 nm, respectively. After measuring the initial fluorescence (F_0_) of the sample, we titrated the labeled cells with sequentially increasing concentrations of ferric siderophore and recorded their corresponding fluorescence (F). We collected data in triplicate, plotted the mean values of 1-F/F_0_ versus [ligand], and analyzed the data with the “1-site with background” binding model of GraFit 6.0.12 (Erithacus Ltd. West Sussex, United Kingdom). The titrations yielded apparent K_D_ values for the binding reactions, as well as fitted curves of [bound ligand] as a function of [free ligand], according to: $\left[\mathrm{Bound}\right]=\frac{\mathrm{Capacity}\cdot \left[\mathrm{Free}\right]}{K_{d}+\left[\mathrm{Free}\right]}+\mathrm{Background}$ .

Throughout this report, affinities are defined by “apparent” K_D_ values, in the sense that we did not directly measure the binding of the iron complexes, but rather the fluorescence quenching that resulted from their binding.

### *In silico* docking

From the structural coordinates of Fiu (PDB ID 6BPM), and a model structure of FeEnt* derived from ferric salmochelin S1 (PubChem CID 135398071). Salmochelin S1 is a degradation product of salmochelin S4 (FeGEnt), lacking one DHBS moiety. After removing its glucose residues, we performed energy minimization using VEGA ZZ 3.2.1. We then used AutoDock Vina (v1.2.1) (Trott and Olson, 2010; Eberhardt et al., 2021) to predict the binding of FeEnt* to Fiu, with the following parameters in the search box: for the whole receptor search, box 1 (size x = 50, y = 78, z = 78; center x = −55, y = 45, z = 20); for binding site 1, box 1 (size x = 24, y = 54, z = 28; center x = −67, y = 36.9, z = 7); for binding site 2, box 2 (size x = 22, y = 56, z = 40; center x = −52, y = 44, z = 4.6); for binding site 3, whole search, box 3 (size x = 50, y = 78, z = 78; center x = −55, y = 45, z = 20). We visualized the top nine ligand poses for each docking run with the AutoDock Tool and Chimera.

### Molecular dynamics simulations

Protein models were prepared by the Schrödinger Protein Preparation Wizard (Sastry et al., 2013). The ligand-docked models originated from the *in silico* ligand docking study, described above. The modeling systems were created by Desmond system builder (Schrödinger Release 2021–4). Each Fiu complex was inserted into a 1-palmitoyl-2-oleoyl-sn-glycero-3-phosphocholine (POPC) lipid bilayer that was perpendicular to the z-axis. SPC water molecules were added into the system; the size of the orthorhombic buffer box was 10 Å x 10 Å x10Å. Sodium chloride (0.15 M) was used to neutralize the system, and we employed OPLS4 (Jorgensen and Tirado-Rives, 1988; Lu et al., 2021) as the force field. Simulation conditions in the isothermal-isobaric (NPT) ensemble were as follows: pressure, 1.01325 bar; temperature, 310 K. Temperature was controlled using a Langevin thermostat. The systems were minimized to 100 ps; the time step was 0.002 ps, and the total production time was 0.5 μs. All data were processed with Schrödinger Desmond. MD simulation movies were generated by Schrödinger Maestro (Release 2021-4).

## Data availability statement

The original contributions presented in the study are included in the article/Supplementary material, further inquiries can be directed to the corresponding author.

## Author contributions

TY: Conceptualization, Data curation, Investigation, Methodology, Writing – original draft, Writing – review & editing, Formal analysis. YZ: Conceptualization, Formal analysis, Investigation, Methodology, Writing – original draft, Writing – review & editing. HN: Conceptualization, Investigation, Methodology, Formal analysis, Supervision, Writing – original draft, Writing – review & editing, Validation. AK: Formal analysis, Investigation, Methodology, Writing – original draft, Validation, Writing – review & editing. SN: Investigation, Methodology, Conceptualization, Formal analysis, Supervision, Validation, Writing – original draft, Writing – original draft, Project administration. PK: Conceptualization, Data curation, Formal analysis, Investigation, Methodology, Supervision, Validation, Project administration, Writing – original draft, Writing – review & editing, Visualization.

## References

[ref1] Abergel R. J. Clifton M. C. Pizarro J. C. Warner J. A. Shuh D. K. Strong R. K.. (2008). The siderocalin/enterobactin interaction: a link between mammalian immunity and bacterial iron transport. J. Am. Chem. Soc. 130, 11524–11534. doi: 10.11510.11021/ja803524w, PMID: 18680288 PMC3188318

[ref2] Ames G. F. (1974). Resolution of bacterial proteins by polyacrylamide gel electrophoresis on slabs. Membrane, soluble, and periplasmic fractions. J. Biol. Chem. 249, 634–644. doi: 10.1016/S0021-9258(19)43074-3, PMID: 4129205

[ref3] Armstrong C. M. Bezanilla F. (1977). Inactivation of the sodium channel. II. Gating current experiments. J. Gen. Physiol. 70, 567–590. doi: 10.1085/jgp.70.5.567, PMID: 591912 PMC2228472

[ref4] Asrat H. Samaroo-Campbell J. Ata S. Quale J. (2023). Contribution of Iron-transport systems and β-lactamases to Cefiderocol resistance in clinical isolates of *Acinetobacter baumannii* endemic to new York City. Antimicrob. Agents Chemother. 67:e0023423. doi: 10.1128/aac.00234-23, PMID: 37212653 PMC10269113

[ref5] Blattner F. R. Plunkett G. III Bloch C. A. Perna N. T. Burland V. Riley M.. (1997). The complete genome sequence of *Escherichia coli* K-12. Science (New York, N.Y.) 277, 1453–1462. doi: 10.1126/science.277.5331.1453, PMID: 9278503

[ref6] Bonhivers M. Ghazi A. Boulanger P. Letellier L. (1996). FhuA, a transporter of the *Escherichia coli* outer membrane, is converted into a channel upon binding of bacteriophage T5. EMBO J. 15, 1850–1856. doi: 10.1002/j.1460-2075.1996.tb00535.x, PMID: 8617231 PMC450102

[ref7] Bradbeer C. (1993). The proton motive force drives the outer membrane transport of cobalamin in *Escherichia coli*. J. Bacteriol. 175, 3146–3150. doi: 10.1128/jb.175.10.3146-3150.1993, PMID: 8387997 PMC204637

[ref8] Bradbeer C. Gudmundsdottir A. (1990). Interdependence of calcium and cobalamin binding by wild-type and mutant BtuB protein in the outer membrane of *Escherichia coli*. J. Bacteriol. 172, 4919–4926. doi: 10.1128/jb.172.9.4919-4926.1990, PMID: 2168369 PMC213146

[ref9] Braun V. Killmann H. Benz R. (1994). Energy-coupled transport through the outer membrane of *Escherichia coli* small deletions in the gating loop convert the FhuA transport protein into a diffusion channel. FEBS Lett. 346, 59–64. doi: 10.1016/0014-5793(94)00431-5, PMID: 7515827

[ref10] Brickman T. J. McIntosh M. A. (1992). Overexpression and purification of ferric enterobactin esterase from *Escherichia coli*. Demonstration of enzymatic hydrolysis of enterobactin and its iron complex. J. Biol. Chem. 267, 12350–12355. doi: 10.1016/S0021-9258(19)49846-3, PMID: 1534808

[ref11] Buchanan S. K. Lukacik P. Grizot S. Ghirlando R. Ali M. M. Barnard T. J.. (2007). Structure of colicin I receptor bound to the R-domain of colicin Ia: implications for protein import. EMBO J. 26, 2594–2604. doi: 10.1038/sj.emboj.7601693, PMID: 17464289 PMC1868905

[ref12] Buchanan S. K. Smith B. S. Venkatramani L. Xia D. Esser L. Palnitkar M.. (1999). Crystal structure of the outer membrane active transporter FepA from *Escherichia coli* [see comments]. Nat. Struct. Biol. 6, 56–63. doi: 10.1038/4931, PMID: 9886293

[ref13] Budzikiewicz H. (2001). Siderophore-antibiotic conjugates used as trojan horses against *Pseudomonas aeruginosa*. Curr. Top. Med. Chem. 1, 73–82. doi: 10.2174/1568026013395524, PMID: 11895295

[ref14] Cadieux N. Phan P. G. Cafiso D. S. Kadner R. J. (2003). Differential substrate-induced signaling through the TonB-dependent transporter BtuB. Proc. Natl. Acad. Sci. USA 100, 10688–10693. doi: 10.1073/pnas.1932538100, PMID: 12958215 PMC196865

[ref15] Cao Z. Warfel P. Newton S. M. Klebba P. E. (2003). Spectroscopic observations of ferric enterobactin transport. J. Biol. Chem. 278, 1022–1028. doi: 10.1074/jbc.M210360200, PMID: 12409288

[ref16] Carmel G. Coulton J. W. (1991). Internal deletions in the FhuA receptor of *Escherichia coli* K-12 define domains of ligand interactions. J. Bacteriol. 173, 4394–4403. doi: 10.1128/jb.173.14.4394-4403.1991, PMID: 2066336 PMC208101

[ref17] Caza M. Garénaux A. Lépine F. Dozois C. M. (2015). Catecholate siderophore esterases Fes, IroD and IroE are required for salmochelins secretion following utilization, but only IroD contributes to virulence of extra-intestinal pathogenic *Escherichia coli*. Mol. Microbiol. 97, 717–732. doi: 10.1111/mmi.13059, PMID: 25982934

[ref18] Chakraborty R. Lemke E. A. Cao Z. Klebba P. E. van der Helm D. (2003). Identification and mutational studies of conserved amino acids in the outer membrane receptor protein, FepA, which affect transport but not binding of ferric-enterobactin in *Escherichia coli*. Biometals 16, 507–518. doi: 10.1023/A:1023485632520 12779236

[ref19] Chakravorty S. Shipelskiy Y. Kumar A. Majumdar A. Yang T. Nairn B. L.. (2019). Universal fluorescent sensors of high-affinity iron transport, applied to ESKAPE pathogens. J. Biol. Chem. 294, 4682–4692. doi: 10.1074/jbc.RA118.006921, PMID: 30679312 PMC6433069

[ref20] Chimento D. P. Mohanty A. K. Kadner R. J. Wiener M. C. (2003). Substrate-induced transmembrane signaling in the cobalamin transporter BtuB. Nat. Struct. Biol. 10, 394–401. doi: 10.1038/nsb914, PMID: 12652322

[ref21] Cobessi D. Celia H. Folschweiller N. Schalk I. J. Abdallah M. A. Pattus F. (2005a). The crystal structure of the pyoverdine outer membrane receptor FpvA from *Pseudomonas aeruginosa* at 3.6 angstroms resolution. J. Mol. Biol. 347, 121–134. doi: 10.1016/j.jmb.2005.01.021, PMID: 15733922

[ref22] Cobessi D. Celia H. Pattus F. (2005b). Crystal structure at high resolution of ferric-pyochelin and its membrane receptor FptA from *Pseudomonas aeruginosa*. J. Mol. Biol. 352, 893–904. doi: 10.1016/j.jmb.2005.08.004, PMID: 16139844

[ref23] Cobessi D. Meksem A. Brillet K. (2010). Structure of the heme/hemoglobin outer membrane receptor ShuA from *Shigella dysenteriae*: heme binding by an induced fit mechanism. Proteins 78, 286–294. doi: 10.1002/prot.22539, PMID: 19731368

[ref24] Cornelissen C. N. Sparling P. F. (1994). Iron piracy: acquisition of transferrin-bound iron by bacterial pathogens. Mol. Microbiol. 14, 843–850. doi: 10.1111/j.1365-2958.1994.tb01320.x, PMID: 7715446

[ref25] Correnti C. Strong R. K. (2012). Mammalian siderophores, siderophore-binding lipocalins, and the labile iron pool. J. Biol. Chem. 287, 13524–13531. doi: 10.1074/jbc.R111.311829, PMID: 22389496 PMC3340207

[ref26] Curtis N. Eisenstadt R. L. East S. J. Cornford R. J. Walker L. A. White A. J. (1988). Iron-regulated outer membrane proteins of *Escherichia coli* K-12 and mechanism of action of catechol-substituted cephalosporins. Antimicrob. Agents Chemother. 32, 1879–1886. doi: 10.1128/AAC.32.12.1879, PMID: 3072926 PMC176037

[ref27] Delgado-Valverde M. Conejo M. D. C. Serrano L. Fernández-Cuenca F. Pascual Á. (2020). Activity of cefiderocol against high-risk clones of multidrug-resistant Enterobacterales, *Acinetobacter baumannii*, Pseudomonas aeruginosa and *Stenotrophomonas maltophilia*. J. Antimicrob. Chemother. 75, 1840–1849. doi: 10.1093/jac/dkaa117, PMID: 32277821 PMC7303814

[ref28] Doneanu C. E. Strong R. K. Howald W. N. (2004). Characterization of a noncovalent lipocalin complex by liquid chromatography/electrospray ionization mass spectrometry. J. Biomol. Tech. 15, 208–212. PMID: 15331587 PMC2291690

[ref29] Eberhardt J. Santos-Martins D. Tillack A. F. Forli S. (2021). AutoDock Vina 1.2.0: new docking methods, expanded force field, and Python bindings. J. Chem. Inf. Model. 61, 3891–3898. doi: 10.1021/acs.jcim.1c00203, PMID: 34278794 PMC10683950

[ref30] Eisenhauer H. A. Shames S. Pawelek P. D. Coulton J. W. (2005). Siderophore transport through *Escherichia coli* outer membrane receptor FhuA with disulfide-tethered cork and barrel domains. J. Biol. Chem. 280, 30574–30580. Epub 32005 Jun 30530. doi: 10.1074/jbc.M506708200, PMID: 15994322

[ref31] Endriss F. Braun M. Killmann H. Braun V. (2003). Mutant analysis of the *Escherichia coli* FhuA protein reveals sites of FhuA activity. J. Bacteriol. 185, 4683–4692. doi: 10.1128/JB.185.16.4683-4692.2003, PMID: 12896986 PMC166461

[ref32] Escalante J. Nishimura B. Tuttobene M. R. Subils T. Mezcord V. Actis L. A.. (2023). The Iron content of human serum albumin modulates the susceptibility of *Acinetobacter baumannii* to Cefiderocol. Biomedicines 11:639. doi: 10.3390/biomedicines11020639 36831178 PMC9953112

[ref33] Fanucci G. E. Cadieux N. Piedmont C. A. Kadner R. J. Cafiso D. S. (2002). Structure and dynamics of the beta-barrel of the membrane transporter BtuB by site-directed spin labeling. Biochemistry 41, 11543–11551. doi: 10.1021/bi0259397, PMID: 12269798

[ref34] Faraldo-Gómez J. D. Smith G. R. Sansom M. S. (2003). Molecular dynamics simulations of the bacterial outer membrane protein FhuA: a comparative study of the ferrichrome-free and bound states. Biophys. J. 85, 1406–1420. doi: 10.1016/S0006-3495(03)74573-1, PMID: 12944258 PMC1303317

[ref35] Ferguson A. D. Chakraborty R. Smith B. S. Esser L. van der Helm D. Deisenhofer J. (2002). Structural basis of gating by the outer membrane transporter FecA. Science (New York, N.Y.) 295, 1715–1719. doi: 10.1126/science.1067313 11872840

[ref36] Ferguson A. D. Hofmann E. Coulton J. W. Diederichs K. Welte W. (1998). Siderophore-mediated iron transport: crystal structure of FhuA with bound lipopolysaccharide [see comments]. Science (New York, N.Y.) 282, 2215–2220. doi: 10.1126/science.282.5397.2215 9856937

[ref37] Fleming I. D. Krezalek M. A. Belogortseva N. Zaborin A. Defazio J. Chandrasekar L.. (2017). Modeling *Acinetobacter baumannii* wound infections: the critical role of iron. J. Trauma Acute Care Surg. 82, 557–565. doi: 10.1097/TA.0000000000001338, PMID: 28030490 PMC5322184

[ref38] Gaddy J. A. Arivett B. A. McConnell M. J. Lopez-Rojas R. Pachon J. Actis L. A. (2012). Role of acinetobactin-mediated iron acquisition functions in the interaction of *Acinetobacter baumannii* strain ATCC 19606T with human lung epithelial cells, galleria mellonella caterpillars, and mice. Infect. Immun. 80, 1015–1024. doi: 10.1128/IAI.06279-11, PMID: 22232188 PMC3294665

[ref39] Grinter R. Lithgow T. (2019a). The structure of the bacterial iron-catecholate transporter Fiu suggests that it imports substrates via a two-step mechanism. J. Biol. Chem. 294, 19523–19534. doi: 10.1074/jbc.RA119.011018, PMID: 31712312 PMC6926462

[ref40] Grinter R. Lithgow T. (2019b). Determination of the molecular basis for coprogen import by gram-negative bacteria. IUCrJ 6, 401–411. doi: 10.1107/S2052252519002926, PMID: 31098021 PMC6503915

[ref41] Grinter R. Lithgow T. (2020). The crystal structure of the TonB-dependent transporter YncD reveals a positively charged substrate-binding site. Acta Crystallogr. D Struct. Biol. 76, 484–495. doi: 10.1107/S2059798320004398, PMID: 32355044 PMC7193533

[ref42] Gudmundsdottir A. Bell P. E. Lundrigan M. D. Bradbeer C. Kadner R. J. (1989). Point mutations in a conserved region (TonB box) of *Escherichia coli* outer membrane protein BtuB affect vitamin B12 transport. J. Bacteriol. 171, 6526–6533. doi: 10.1128/jb.171.12.6526-6533.1989, PMID: 2687240 PMC210543

[ref43] Gumbart J. C. Wiener M. C. Tajkhorshid E. (2007). Mechanics of force propagation in TonB-dependent outer membrane transport. Biophys. J. 93, 496–504. doi: 10.1529/biophysj.107.104158, PMID: 17449669 PMC1896255

[ref44] Gupta A. Landman D. Quale J. (2022). Relationship of TonB-dependent receptors with susceptibility to cefiderocol in clinical isolates of *Pseudomonas aeruginosa*. J. Antimicrob. Chemother. 77, 1282–1285. doi: 10.1093/jac/dkac022, PMID: 35134942

[ref45] Hancock R. E. Braun V. (1976). The colicin I receptor of *Escherichia coli* K-12 has a role in enterochelin-mediated iron transport. FEBS Lett. 65, 208–210. doi: 10.1016/0014-5793(76)80481-4, PMID: 776694

[ref46] Hanson M. Jordan L. D. Shipelskiy Y. Newton S. M. Klebba P. E. (2016). High-throughput screening assay for inhibitors of TonB-dependent Iron transport. J. Biomol. Screen. 21, 316–322. doi: 10.1177/1087057115613788, PMID: 26518031

[ref47] Hantke K. (1983). Identification of an iron uptake system specific for coprogen and rhodotorulic acid in *Escherichia coli* K12. Mol. Gen. Genet. 191, 301–306. doi: 10.1007/BF00334830, PMID: 6353165

[ref48] Hantke K. (1990). Dihydroxybenzoylserine--a siderophore for *E. coli*. FEMS Microbiol. Lett. 55, 5–8. doi: 10.1016/0378-1097(90)90158-m, PMID: 2139424

[ref49] Hantke K. Nicholson G. Rabsch W. Winkelmann G. (2003). Salmochelins, siderophores of salmonella enterica and uropathogenic *Escherichia coli* strains, are recognized by the outer membrane receptor IroN. Proc. Natl. Acad. Sci. USA 100, 3677–3682. doi: 10.1073/pnas.0737682100, PMID: 12655053 PMC152981

[ref50] Harris W. R. Carrano C. J. Raymond K. N. (1979). Spectrophotometric determination of the proton-dependent stability constant of ferric enterobactin. J. Am. Chem. Soc. 101, 2213–2214. doi: 10.1021/ja00502a053

[ref51] Holmes M. A. Paulsene W. Jide X. Ratledge C. Strong R. K. (2005). Siderocalin (Lcn 2) also binds carboxymycobactins, potentially defending against mycobacterial infections through iron sequestration. Structure 13, 29–41. doi: 10.1016/j.str.2004.10.009 15642259

[ref52] Hunter M. G. Glass R. E. (1982). Analysis of btuB receptor function by use of nonsense suppression. J. Bacteriol. 151, 1591–1594. doi: 10.1128/jb.151.3.1591-1594.1982, PMID: 7050093 PMC220441

[ref53] Ito A. I. N. Ishii R. Tsuji M. Maki H. Sato T. Yamano Y. (2019). “Changes of responsible Iron-transporters for the activity of Cefiderocol against *Pseudomonas aeruginosa* depending on the culture conditions” in ASM Microbe (San Francisco, CA: ASM.)

[ref54] Ito A. Sato T. Ota M. Takemura M. Nishikawa T. Toba S.. (2018). In vitro antibacterial properties of cefiderocol, a novel siderophore cephalosporin, against gram-negative bacteria. Antimicrob. Agents Chemother. 62, e01454–e01417. doi: 10.1128/AAC.01454-17 PMC5740388 29061741

[ref55] Ji C. Juárez-Hernández R. E. Miller M. J. (2012). Exploiting bacterial iron acquisition: siderophore conjugates. Future Med. Chem. 4, 297–313. doi: 10.4155/fmc.11.191, PMID: 22393938 PMC6901097

[ref56] Jiang X. Payne M. A. Cao Z. Foster S. B. Feix J. B. Newton S. M.. (1997). Ligand-specific opening of a gated-porin channel in the outer membrane of living bacteria. Science (New York, N.Y.) 276, 1261–1264. doi: 10.1126/science.276.5316.1261 9157886

[ref57] Johnson J. R. Russo T. A. Scheutz F. Brown J. J. Zhang L. Palin K.. (1997). Discovery of disseminated J96-like strains of uropathogenic *Escherichia coli* O4: H5 containing genes for both PapGJ96 (class I) and PrsGJ96 (class III) gal (α1-4) gal-binding adhesins. J. Infect. Dis. 175, 983–988. doi: 10.1086/514006, PMID: 9086165

[ref58] Jordan L. D. Zhou Y. Smallwood C. R. Lill Y. Ritchie K. Yip W. T.. (2013). Energy-dependent motion of TonB in the gram-negative bacterial inner membrane. Proc. Natl. Acad. Sci. USA 110, 11553–11558. doi: 10.1073/pnas.1304243110, PMID: 23798405 PMC3710835

[ref59] Jorgensen W. L. Tirado-Rives J. (1988). The OPLS [optimized potentials for liquid simulations] potential functions for proteins, energy minimizations for crystals of cyclic peptides and crambin. J. Am. Chem. Soc. 110, 1657–1666. doi: 10.1021/ja00214a001, PMID: 27557051

[ref60] Kim A. Kutschke A. Ehmann D. E. Patey S. A. Crandon J. L. Gorseth E.. (2015). Pharmacodynamic profiling of a siderophore-conjugated monocarbam in *Pseudomonas aeruginosa*: assessing the risk for resistance and attenuated efficacy. Antimicrob. Agents Chemother. 59, 7743–7752. doi: 10.1128/AAC.00831-15, PMID: 26438502 PMC4649189

[ref61] Klebba P. E. (1981) Regulation of the bisynthesis of the iron-related membrane proteins in *Escherichia coli*.

[ref62] Klebba P. E. (2003). Three paradoxes of ferric enterobactin uptake. Front. Biosci. 8, s1422–s1436. doi: 10.2741/1233 12957833

[ref63] Klebba P. E. (2016). ROSET model of TonB action in gram-negative bacterial Iron acquisition. J. Bacteriol. 198, 1013–1021. doi: 10.1128/JB.00823-15, PMID: 26787763 PMC4800874

[ref64] Klebba P. E. McIntosh M. A. Neilands J. B. (1982). Kinetics of biosynthesis of iron-regulated membrane proteins in *Escherichia coli*. J. Bacteriol. 149, 880–888. doi: 10.1128/jb.149.3.880-888.1982, PMID: 6174499 PMC216474

[ref65] Klebba P. E. Newton S. M. Six D. A. Kumar A. Yang T. Nairn B. L.. (2021). Iron acquisition systems of gram (−) bacterial pathogens define TonB-dependent pathways to novel antibiotics. Chem. Rev. 121, 5193–5239. doi: 10.1021/acs.chemrev.0c01005 33724814 PMC8687107

[ref66] Kumar A. Yang T. Chakravorty S. Majumdar A. Nairn B. L. Six D. A.. (2022). Fluorescent sensors of siderophores produced by bacterial pathogens. J. Biol. Chem. 298:101651. doi: 10.1016/j.jbc.2022.101651, PMID: 35101443 PMC8921320

[ref67] Lasko M. J. Nicolau D. P. (2020). Carbapenem-resistant Enterobacterales: considerations for treatment in the era of new antimicrobials and evolving enzymology. Curr. Infect. Dis. Rep. 22:6. doi: 10.1007/s11908-020-0716-3, PMID: 32034524 PMC7223591

[ref68] Le C. Pimentel C. Pasteran F. Tuttobene M. R. Subils T. Escalante J.. (2022). Human serum proteins and susceptibility of *Acinetobacter baumannii* to Cefiderocol: role of Iron transport. Biomedicines 10:600. doi: 10.3390/biomedicines10030600, PMID: 35327400 PMC8945497

[ref69] Lin H. Fischbach M. A. Liu D. R. Walsh C. T. (2005). In vitro characterization of salmochelin and enterobactin trilactone hydrolases IroD, IroE, and Fes. J. Am. Chem. Soc. 127, 11075–11084. doi: 10.1021/ja0522027, PMID: 16076215 PMC2536649

[ref70] Locher K. P. Rees B. Koebnik R. Mitschler A. Moulinier L. Rosenbusch J. P.. (1998). Transmembrane signaling across the ligand-gated FhuA receptor: crystal structures of free and ferrichrome-bound states reveal allosteric changes. Cell 95, 771–778. doi: 10.1016/S0092-8674(00)81700-6, PMID: 9865695

[ref71] Lu C. Wu C. Ghoreishi D. Chen W. Wang L. Damm W.. (2021). OPLS4: improving force field accuracy on challenging regimes of chemical space. J. Chem. Theory Comput. 17, 4291–4300. doi: 10.1021/acs.jctc.1c00302, PMID: 34096718

[ref72] Lugtenberg B. Meijers J. Peters R. van der Hoek P. van Alphen L. (1975). Electrophoretic resolution of the "major outer membrane protein" of *Escherichia coli* K12 into four bands. FEBS Lett. 58, 254–258. doi: 10.1016/0014-5793(75)80272-9 773686

[ref73] Luna B. M. Ershova K. Yan J. Ulhaq A. Nielsen T. B. Hsieh S.. (2019). Adjunctive transferrin to reduce the emergence of antibiotic resistance in gram-negative bacteria. J. Antimicrob. Chemother. 74, 2631–2639. doi: 10.1093/jac/dkz225, PMID: 31170282 PMC6736376

[ref74] Luscher A. Moynié L. Auguste P. S. Bumann D. Mazza L. Pletzer D.. (2018). TonB-dependent receptor repertoire of *Pseudomonas aeruginosa* for uptake of Siderophore-drug conjugates. Antimicrob. Agents Chemother. 62:e00097-18. doi: 10.1128/AAC.00097-18, PMID: 29555629 PMC5971595

[ref75] Ma L. Kaserer W. Annamalai R. Scott D. C. Jin B. Jiang X.. (2007). Evidence of ball-and-chain transport of ferric enterobactin through FepA. J. Biol. Chem. 282, 397–406. doi: 10.1074/jbc.M605333200, PMID: 17056600 PMC2398697

[ref76] Mabayoje D. A. NicFhogartaigh C. Cherian B. P. Tan M. G. M. Wareham D. W. (2021). Compassionate use of cefiderocol for carbapenem-resistant *Acinetobacter baumannii* prosthetic joint infection. JAC Antimicrob. Resist. 3, i21–i24. doi: 10.1093/jacamr/dlab055, PMID: 34223152 PMC8251250

[ref77] Majumdar A. Trinh V. Moore K. J. Smallwood C. R. Kumar A. Yang T.. (2020). Conformational rearrangements in the N-domain of *Escherichia coli* FepA during ferric enterobactin transport. J. Biol. Chem. 295, 4974–4984. doi: 10.1074/jbc.RA119.011850, PMID: 32098871 PMC7152776

[ref78] Malik S. Kaminski M. Landman D. Quale J. (2020). Cefiderocol resistance in *Acinetobacter baumannii*: roles of β-lactamases, Siderophore receptors, and penicillin binding protein 3. Antimicrob. Agents Chemother. 64:e01221-20. doi: 10.1128/AAC.01221-20, PMID: 32868330 PMC7577126

[ref79] McCreary E. K. Heil E. L. Tamma P. D. (2021). New perspectives on antimicrobial agents: Cefiderocol. Antimicrob. Agents Chemother. 65:e0217120. doi: 10.1128/AAC.02171-20, PMID: 34031052 PMC8373209

[ref80] McIntosh M. A. Earhart C. F. (1976). Effect of iron of the relative abundance of two large polypeptides of the *Escherichia coli* outer membrane. Biochem. Biophys. Res. Commun. 70, 315–322. doi: 10.1016/0006-291X(76)91144-X, PMID: 776187

[ref81] Miller M. J. Liu R. (2021). Design and syntheses of new antibiotics inspired by Nature's quest for Iron in an oxidative climate. Acc. Chem. Res. 54, 1646–1661. doi: 10.1021/acs.accounts.1c00004, PMID: 33684288 PMC9262095

[ref82] Miller M. J. McKee J. A. Minnick A. A. Dolence E. K. (1991). The design, synthesis and study of siderophore-antibiotic conjugates. Siderophore mediated drug transport. Biol. Met. 4, 62–69. doi: 10.1007/BF01135559, PMID: 1830210

[ref83] Moeck G. S. Coulton J. W. Postle K. (1997). Cell envelope signaling in *Escherichia coli*. Ligand binding to the ferrichrome-iron receptor fhua promotes interaction with the energy- transducing protein TonB. J. Biol. Chem. 272, 28391–28397. doi: 10.1074/jbc.272.45.28391 9353297

[ref84] Morris C. P. Bergman Y. Tekle T. Fissel J. A. Tamma P. D. Simner P. J. (2020). Cefiderocol antimicrobial susceptibility testing against multidrug-resistant gram-negative Bacilli: a comparison of disk diffusion to broth microdilution. J. Clin. Microbiol. 59:e01649-20. doi: 10.1128/JCM.01649-20, PMID: 32938734 PMC7771458

[ref85] Nairn B. L. Eliasson O. S. Hyder D. R. Long N. J. Majumdar A. Chakravorty S.. (2017). Fluorescence high-throughput screening for inhibitors of TonB action. J. Bacteriol. 199:e00889-16. doi: 10.1128/JB.00889-16 28242720 PMC5405212

[ref86] Nazareth H. Genagon S. A. Russo T. A. (2007). Extraintestinal pathogenic *Escherichia coli* survives within neutrophils. Infect. Immun. 75, 2776–2785. doi: 10.1128/IAI.01095-06, PMID: 17296761 PMC1932911

[ref87] Neidhardt F. C. Bloch P. L. Smith D. F. (1974). Culture medium for enterobacteria. J. Bacteriol. 119, 736–747. doi: 10.1128/jb.119.3.736-747.1974, PMID: 4604283 PMC245675

[ref88] Neilands J. (1974). “Iron and its role in microbial physiology” in Microbial iron metabolism. ed. Neilands J. B. (Cambridge, MA: Academic Press), 3–34.

[ref89] Neilands J. B. (1995). Siderophores: structure and function of microbial iron transport compounds. J. Biol. Chem. 270, 26723–26726. doi: 10.1074/jbc.270.45.26723, PMID: 7592901

[ref90] Newton S. M. Allen J. S. Cao Z. Qi Z. Jiang X. Sprencel C.. (1997). Double mutagenesis of a positive charge cluster in the ligand-binding site of the ferric enterobactin receptor, FepA. Proc. Natl. Acad. Sci. USA 94, 4560–4565. doi: 10.1073/pnas.94.9.4560, PMID: 9114029 PMC20762

[ref91] Newton S. M. Igo J. D. Scott D. C. Klebba P. E. (1999). Effect of loop deletions on the binding and transport of ferric enterobactin by FepA. Mol. Microbiol. 32, 1153–1165. doi: 10.1046/j.1365-2958.1999.01424.x, PMID: 10383757

[ref92] Newton S. M. Klebba P. E. (2022). Fluorescent binding protein sensors for detection and quantification of biochemicals, metabolites, and natural products. Bio Protoc. 12:e4543. doi: 10.21769/BioProtoc.4543, PMID: 36532683 PMC9724013

[ref93] Newton S. M. Klebba P. E. Raynaud C. Shao Y. Jiang X. Dubail I.. (2005). The svpA-srtB locus of *Listeria monocytogenes*: Fur-mediated iron regulation and effect on virulence. Mol. Microbiol. 55, 927–940. doi: 10.1111/j.1365-2958.2004.04436.x, PMID: 15661014

[ref94] Newton S. M. Trinh V. Pi H. Klebba P. E. (2010). Direct measurements of the outer membrane stage of ferric enterobactin transport: postuptake binding. J. Biol. Chem. 285, 17488–17497. doi: 10.1074/jbc.M109.100206, PMID: 20335169 PMC2878513

[ref95] Nikaido H. Rosenberg E. Y. (1990). Cir and Fiu proteins in the outer membrane of *Escherichia coli* catalyze transport of monomeric catechols: study with beta-lactam antibiotics containing catechol and analogous groups. J. Bacteriol. 172, 1361–1367. doi: 10.1128/jb.172.3.1361-1367.1990, PMID: 2407721 PMC208606

[ref96] Noinaj N. Guillier M. Barnard T. J. Buchanan S. K. (2010). TonB-dependent transporters: regulation, structure, and function. Ann. Rev. Microbiol. 64, 43–60. doi: 10.1146/annurev.micro.112408.134247, PMID: 20420522 PMC3108441

[ref97] Nyenhuis D. A. Nilaweera T. D. Cafiso D. S. (2020). Native cell environment constrains loop structure in the *Escherichia coli* cobalamin transporter BtuB. Biophys. J. 119, 1550–1557. doi: 10.1016/j.bpj.2020.08.034, PMID: 32946767 PMC7642247

[ref98] Pawelek P. D. Croteau N. Ng-Thow-Hing C. Khursigara C. M. Moiseeva N. Allaire M.. (2006). Structure of TonB in complex with FhuA, *E. coli* outer membrane receptor. Science 312, 1399–1402. doi: 10.1126/science.1128057, PMID: 16741125

[ref99] Payne M. A. Igo J. D. Cao Z. Foster S. B. Newton S. M. Klebba P. E. (1997). Biphasic binding kinetics between FepA and its ligands. J. Biol. Chem. 272, 21950–21955. doi: 10.1074/jbc.272.35.21950, PMID: 9268330

[ref100] Penwell W. F. DeGrace N. Tentarelli S. Gauthier L. Gilbert C. M. Arivett B. A.. (2015). Discovery and characterization of new Hydroxamate Siderophores, Baumannoferrin A and B, produced by *Acinetobacter baumannii*. Chembiochem 16, 1896–1904. doi: 10.1002/cbic.201500147 26235845

[ref101] Ramirez M. S. Penwell W. F. Traglia G. M. Zimbler D. L. Gaddy J. A. Nikolaidis N.. (2019). Identification of potential virulence factors in the model strain *Acinetobacter baumannii* A118. Front. Microbiol. 10:1599. doi: 10.3389/fmicb.2019.01599, PMID: 31396168 PMC6663985

[ref102] Russo T. A. Guenther J. E. Wenderoth S. Frank M. M. (1993). Generation of isogenic K54 capsule-deficient *Escherichia coli* strains through TnphoA-mediated gene disruption. Mol. Microbiol. 9, 357–364. doi: 10.1111/j.1365-2958.1993.tb01696.x, PMID: 8412686

[ref103] Russo T. A. McFadden C. D. Carlino-MacDonald U. B. Beanan J. M. Barnard T. J. Johnson J. R. (2002). IroN functions as a siderophore receptor and is a urovirulence factor in an extraintestinal pathogenic isolate of *Escherichia coli*. Infect. Immun. 70, 7156–7160. doi: 10.1128/IAI.70.12.7156-7160.2002, PMID: 12438401 PMC133021

[ref104] Russo T. A. Olson R. MacDonald U. Beanan J. Davidson B. A. (2015). Aerobactin, but not yersiniabactin, salmochelin, or enterobactin, enables the growth/survival of hypervirulent (hypermucoviscous) *Klebsiella pneumoniae* ex vivo and in vivo. Infect. Immun. 83, 3325–3333. doi: 10.1128/IAI.00430-15, PMID: 26056379 PMC4496593

[ref105] Russo T. A. Shon A. S. Beanan J. M. Olson R. MacDonald U. Pomakov A. O.. (2011). Hypervirulent *K. pneumoniae* secretes more and more active iron-acquisition molecules than "classical" *K. pneumoniae* thereby enhancing its virulence. PLoS One 6:e26734. doi: 10.26710.21371/journal.pone.0026734, PMID: 22039542 PMC3200348

[ref106] Rutz J. M. Liu J. Lyons J. A. Goranson J. Armstrong S. K. McIntosh M. A.. (1992). Formation of a gated channel by a ligand-specific transport protein in the bacterial outer membrane. Science (New York, N.Y.) 258, 471–475. doi: 10.1126/science.1411544 1411544

[ref107] Sastry G. M. Adzhigirey M. Day T. Annabhimoju R. Sherman W. (2013). Protein and ligand preparation: parameters, protocols, and influence on virtual screening enrichments. J. Comput. Aided Mol. Des. 27, 221–234. doi: 10.1007/s10822-013-9644-8, PMID: 23579614

[ref108] Schauer K. Rodionov D. A. de Reuse H. (2008). New substrates for TonB-dependent transport: do we only see the 'tip of the iceberg'? Trends Biochem. Sci. 33, 330–338. doi: 10.1016/j.tibs.2008.04.012 18539464

[ref109] Scott D. C. Newton S. M. Klebba P. E. (2002). Surface loop motion in FepA. J. Bacteriol. 184, 4906–4911. doi: 10.1128/JB.184.17.4906-4911.2002, PMID: 12169616 PMC135268

[ref110] Shea C. M. McIntosh M. A. (1991). Nucleotide sequence and genetic organization of the ferric enterobactin transport system: homology to other periplasmic binding protein- dependent systems in *Escherichia coli*. Mol. Microbiol. 5, 1415–1428. doi: 10.1111/j.1365-2958.1991.tb00788.x, PMID: 1838574

[ref111] Shields R. K. (2020). Case commentary: the need for Cefiderocol is clear, but are the supporting clinical data? Antimicrob. Agents Chemother. 64:e00059-20. doi: 10.1128/AAC.00059-20 32015037 PMC7179324

[ref112] Shultis D. D. Purdy M. D. Banchs C. N. Wiener M. C. (2006). Outer membrane active transport: structure of the BtuB:TonB complex. Science 312, 1396–1399. doi: 10.1126/science.1127694, PMID: 16741124

[ref113] Simner P. J. Patel R. (2020). Cefiderocol antimicrobial susceptibility testing considerations: the Achilles' heel of the Trojan horse? J. Clin. Microbiol. 59:e00951-20. doi: 10.1128/JCM.00951-20, PMID: 32727829 PMC7771437

[ref114] Smallwood C. R. Jordan L. Trinh V. Schuerch D. W. Gala A. Hanson M.. (2014). Concerted loop motion triggers induced fit of FepA to ferric enterobactin. J. Gen. Physiol. 144, 71–80. doi: 10.1085/jgp.201311159, PMID: 24981231 PMC4076525

[ref115] Smoke S. M. Brophy A. Reveron S. Iovleva A. Kline E. G. Marano M.. (2023). Evolution and transmission of Cefiderocol-resistant *Acinetobacter baumannii* during an outbreak in the burn intensive care unit. Clin. Infect. Dis. 76, e1261–e1265. doi: 10.1093/cid/ciac647, PMID: 35974429 PMC10169418

[ref116] Sprencel C. Cao Z. Qi Z. Scott D. C. Montague M. A. Ivanoff N.. (2000). Binding of ferric enterobactin by the *escherichia coli* periplasmic protein fepB. J. Bacteriol. 182, 5359–5364. doi: 10.1128/JB.182.19.5359-5364.2000, PMID: 10986237 PMC110977

[ref117] Takeshita S. Sato M. Toba M. Masahashi W. Hashimoto-Gotoh T. (1987). High-copy-number and low-copy-number plasmid vectors for lacZα-complementation and chloramphenicol-or kanamycin-resistance selection. Gene 61, 63–74. doi: 10.1016/0378-1119(87)90365-9 3327753

[ref118] Thulasiraman P. Newton S. M. Xu J. Raymond K. N. Mai C. Hall A.. (1998). Selectivity of ferric enterobactin binding and cooperativity of transport in gram-negative bacteria. J. Bacteriol. 180, 6689–6696. doi: 10.1128/JB.180.24.6689-6696.1998, PMID: 9852016 PMC107775

[ref119] Tiseo G. Giordano C. Leonildi A. Riccardi N. Galfo V. Limongi F.. (2023). Salvage therapy with sulbactam/durlobactam against cefiderocol-resistant *Acinetobacter baumannii* in a critically ill burn patient: clinical challenges and molecular characterization. JAC Antimicrob. Resist. 5:dlad078. doi: 10.1093/jacamr/dlad078, PMID: 37325251 PMC10265591

[ref120] Trott O. Olson A. J. (2010). AutoDock Vina: improving the speed and accuracy of docking with a new scoring function, efficient optimization, and multithreading. J. Comput. Chem. 31, 455–461. doi: 10.1002/jcc.21334, PMID: 19499576 PMC3041641

[ref121] Wayne R. Frick K. Neilands J. B. (1976). Siderophore protection against colicins M, B, V, and Ia in *Escherichia coli*. J. Bacteriol. 126, 7–12. doi: 10.1128/jb.126.1.7-12.1976, PMID: 131121 PMC233253

[ref122] Wayne R. Neilands J. B. (1975). Evidence for common binding sites for ferrichrome compounds and bacteriophage phi 80 in the cell envelope of *Escherichia coli*. J. Bacteriol. 121, 497–503. doi: 10.1128/jb.121.2.497-503.1975, PMID: 803957 PMC245958

[ref123] Wencewicz T. A. Long T. E. Mollmann U. Miller M. J. (2013). Trihydroxamate siderophore-fluoroquinolone conjugates are selective sideromycin antibiotics that target *Staphylococcus aureus*. Bioconjug. Chem. 24, 473–486. doi: 10.410.1021/bc300610f, PMID: 23350642 PMC3633530

[ref124] Wu J. Y. Srinivas P. Pogue J. M. (2020). Cefiderocol: A novel agent for the management of multidrug-resistant gram-negative organisms. Infect. Dis. Ther. 9, 17–40. doi: 10.1007/s40121-020-00286-6, PMID: 32072491 PMC7054475

[ref125] Yamano Y. (2019). In vitro activity of Cefiderocol against a broad range of clinically important gram-negative Bacteria. Clin. Infect. Dis. 69, S544–S551. doi: 10.1093/cid/ciz827, PMID: 31724049 PMC6853761

[ref126] Zhanel G. G. Golden A. R. Zelenitsky S. Wiebe K. Lawrence C. K. Adam H. J.. (2019). Cefiderocol: A Siderophore cephalosporin with activity against Carbapenem-resistant and multidrug-resistant gram-negative Bacilli. Drugs 79, 271–289. doi: 10.1007/s40265-019-1055-2, PMID: 30712199

[ref127] Zhu M. Valdebenito M. Winkelmann G. Hantke K. (2005). Functions of the siderophore esterases IroD and IroE in iron-salmochelin utilization. Microbiology (Reading) 151, 2363–2372. doi: 10.1099/mic.0.27888-0, PMID: 16000726

