# Supplementary material for: Specificity and mechanism of TonB-dependent ferric catecholate uptake by Fiu
Source: Front Microbiol. 2024 Mar 27;15:1355253. doi: 10.3389/fmicb.2024.1355253 (PMC11005823; doi:10.3389/fmicb.2024.1355253)
Supplement: Supplementary file 2 [file Data_Sheet_1.PDF]

Supplemental Information

Specificity and Mechanism of TonB-dependent Ferric Catecholate Uptake by Fiu

Taihao Yang[1], Ye Zou[1], Ho Leung Ng[1], Ashish Kumar[1,] Salete M. Newton[1] and Phillip E. Klebba[1]*

[1]Department of Biochemistry & Molecular Biophysics, Kansas State University, Manhattan, KS 66506

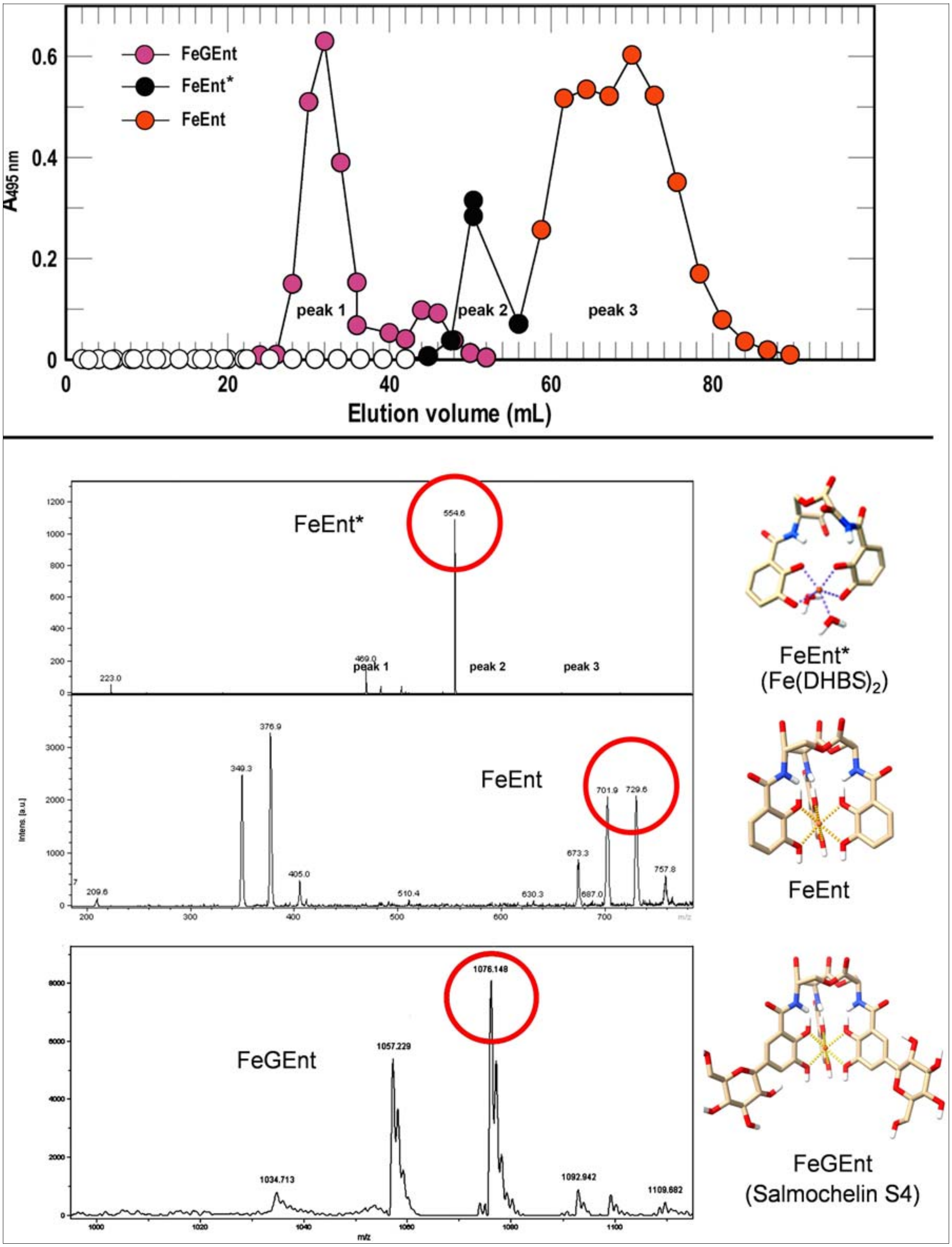

Figure S1. Mass spectrometry of ferric catecholates purified from *E. coli* CP9. We purified FeEnt, FeEnt* and FeGEnt from T-media supernatants of CP9. We first adsorbed the negatively charged chelates in the ferrated supernatant to DE52 ion-exchange cellulose in 50 mM Tris-HCl, pH7, washed the column with 5 volumes of the same buffer, and eluted the ferric siderophores with gradient of ammonium chloride. **(A)** After desalting the eluted peaks on Sephadex G10 in 5 mM $NaHPO_4$, pH 7, we further purified them by gel filtration on Sephadex LH20 in 5 mM $NaHPO_4$, pH 7. We identified the three red-purple fractions of FeEnt, FeEnt* and FeGEnt by their visible absorbance spectra, with unique minima/maxima at 393/495, 405/505 and 435/520 nm, respectively (7). **(B)** We next determined their molecular masses by mass spectrometry [(A) FeEnt*; (B) FeEnt; (C) FeGEnt] and used the purified compounds in siderophore nutrition tests and fluorescence spectroscopic experiments.

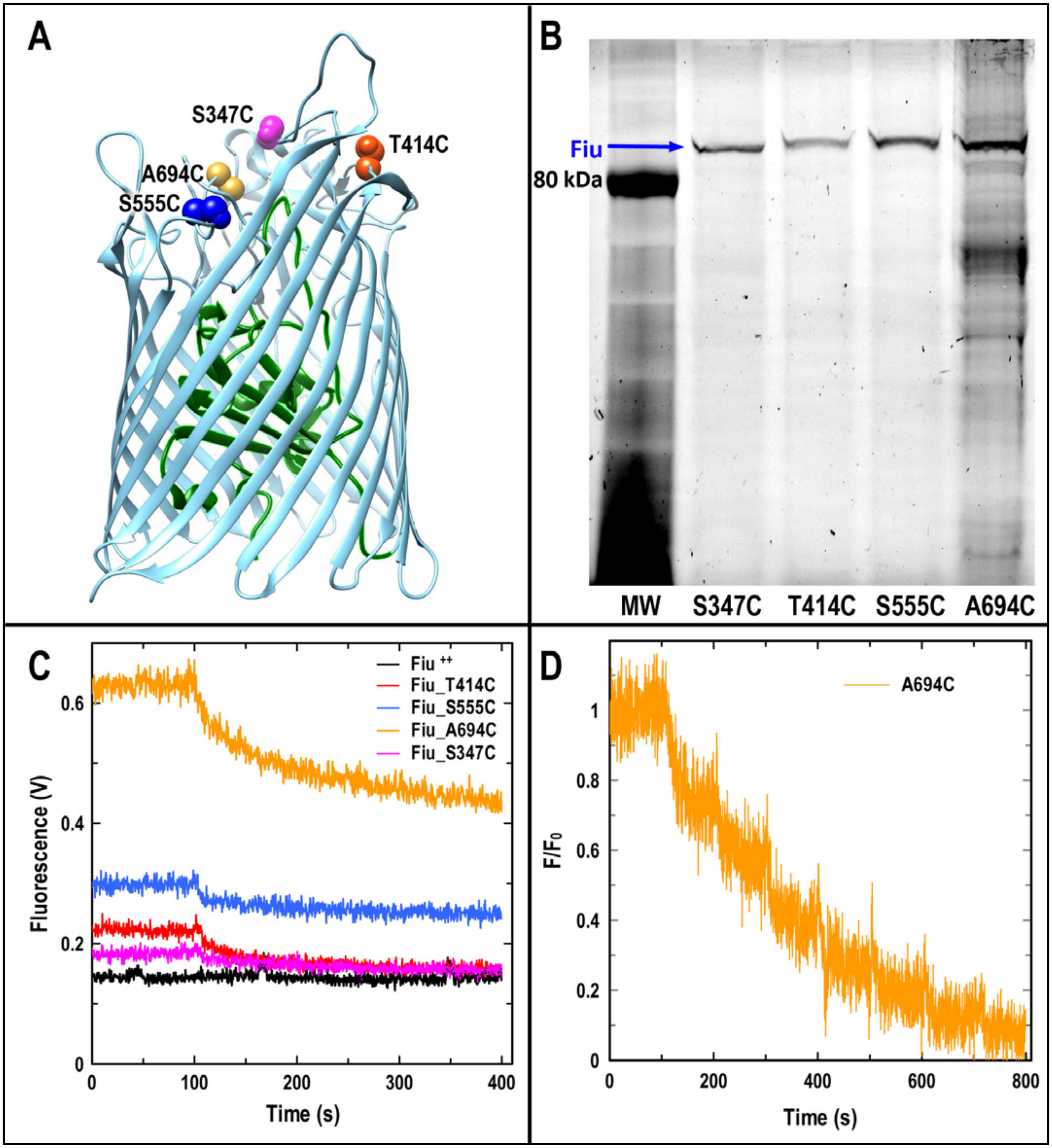

Figure S2. Affinity determinations by fluorescence quenching. (**A)** After selecting a few residues in the loops of Fiu and engineering their substitution by Cys, we performed fluorescent scans of SDS-PAGE gels of their OM fractions. (**B**). FM specifically modified all four Cys mutants. (**C**) Spectroscopic analyses of cells expressing the same Cys mutants showed that Fiu_A694C-FM (orange tracing) was the brightest derivative, and the most sensitive to quenching by 0.7 uM FeEnt*. Note that wild-type Fiu (black) was neither strongly labeled by FM, nor responsive to the addition of FeEnt*. Cells expressing wild-type Fiu (black tracing) were labeled at background levels, and insensitive to the addition of FeEnt*. (**D**) Exposure of OKN1359/pFiu_A694C-FM (5x10^7 cells/mL) to increasing concentrations of FeEnt* led to a quenching time course that revealed the binding affinity.

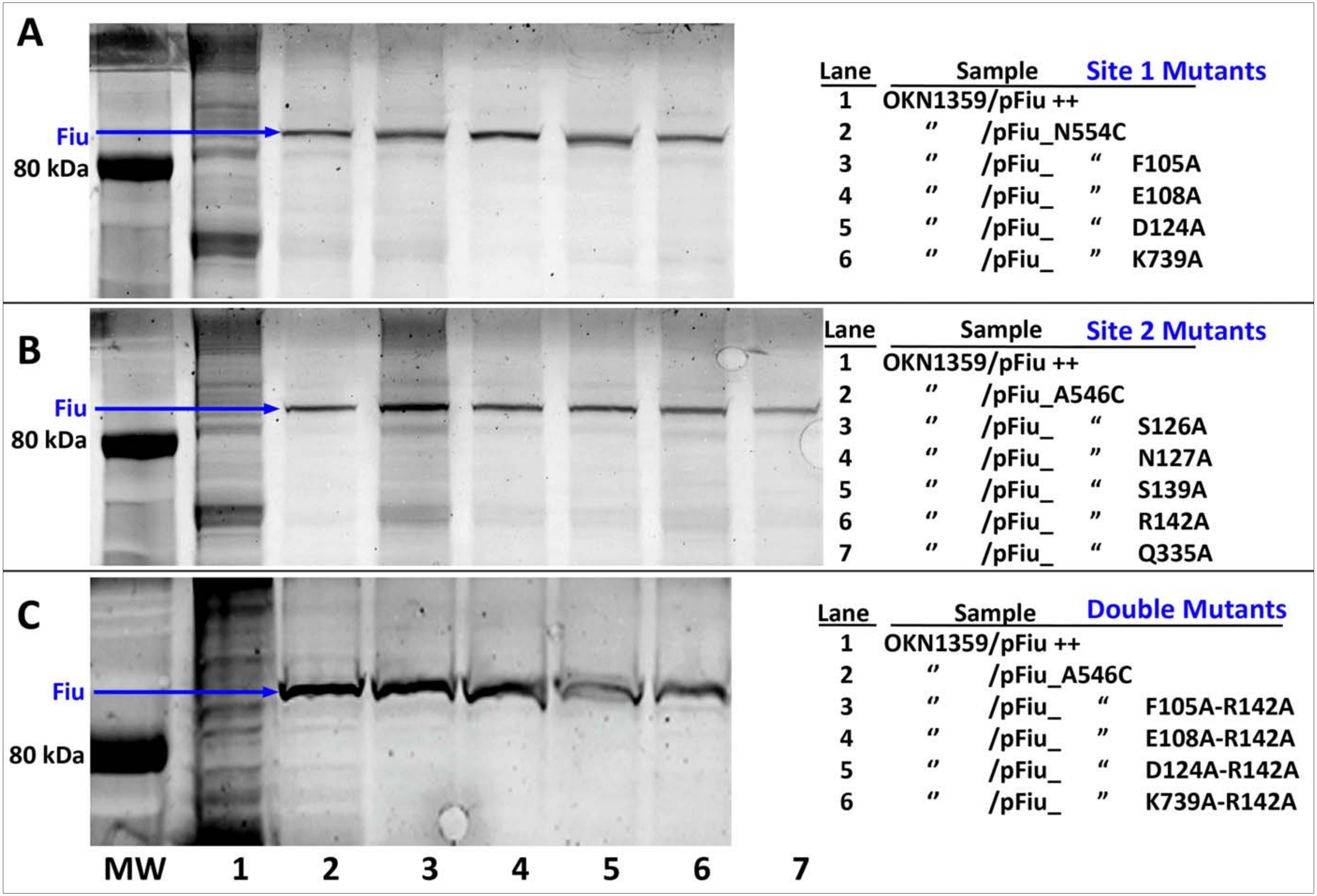

Figure S3. Fluorescence imaging of FM-labeled OM fractions from Fiu Cys/Ala substitution mutants. The SDS-PAGE gels show the specific labeling of Fiu_N554C in site 1 (A), and A546C in site 2 (B), before and after combining the Cys mutants with Ala substitutions for residues of interest in those binding sites. The labeling of double mutants in both sites (C) was similarly efficient.

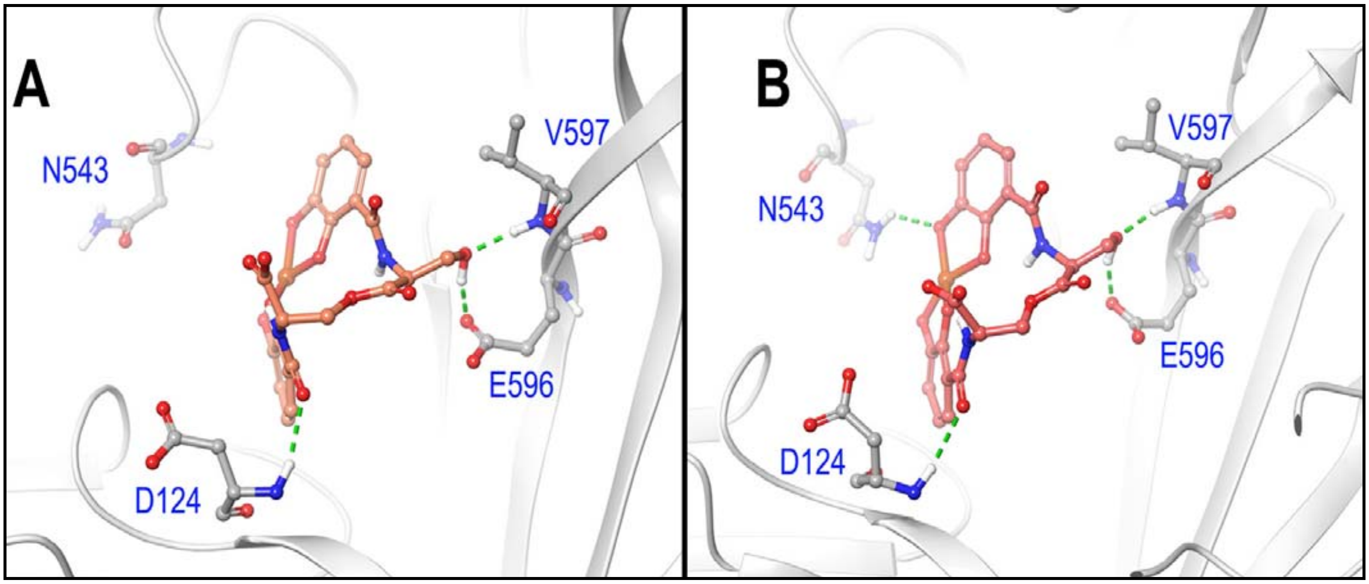

Figure S4. Transition States. The movement of ligand from 1a to 1b occurred through two transition states. **A. Transition State 1.** From its initial location, bound by F105, D124 and K739 in site 1a, FeEnt* separated from F105 and H-bonded to E596 and V597, while retaining its H-bond to D124. **B. Transition State 2.** The ligand next H-bonded to N543, while retaining its H-bonds with D124, E596 and V597. FeEnt* then separated from E596 and V597 and formed a new H-bond to N127 in site 1b. Ultimately, FeEnt* interacted with D124, N127 and N543 in site 1b through H-bonds. D124 remained associated with FeEnt* throughout this reorganization from site 1a, through the transition states, and into site 1b.

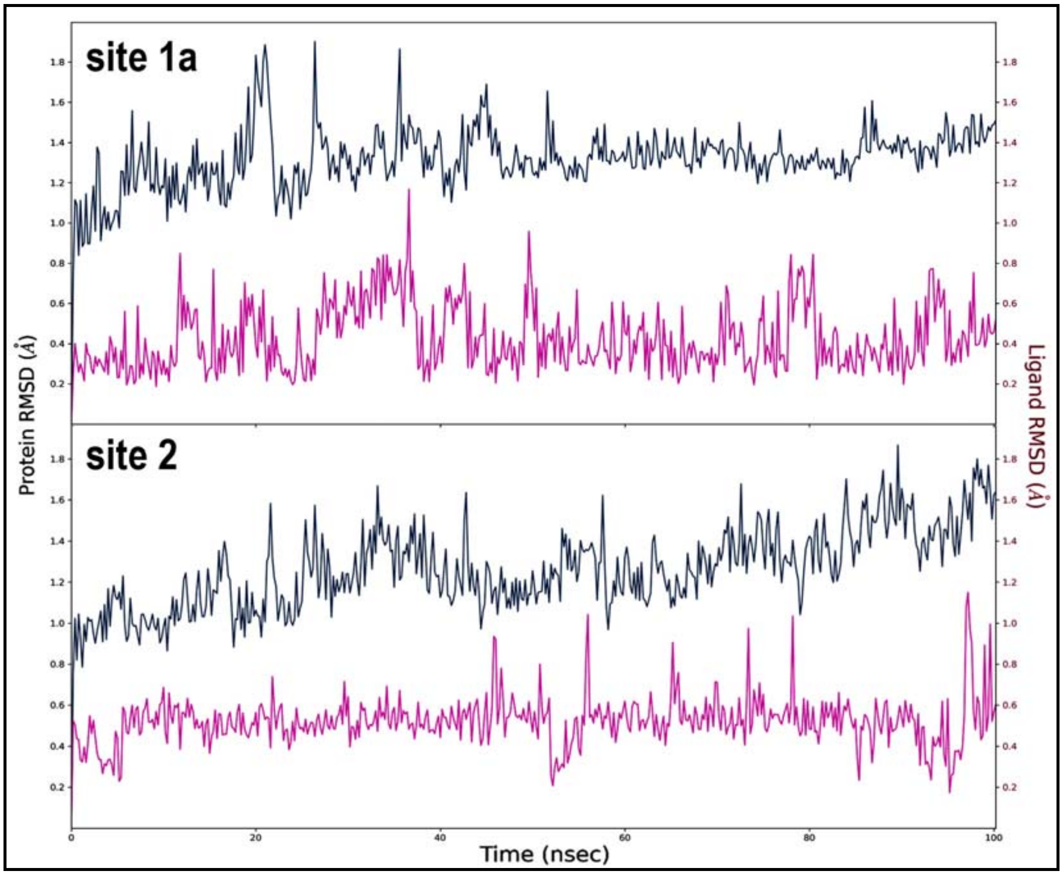

Figure S5. MD simulation over 100 nanoseconds. Protein and Ligand Root Mean Square Deviation (RMSD) fluctuations in sites 1a and 2 over the last 100 ns of the simulation (total simulation time was 500 ns).

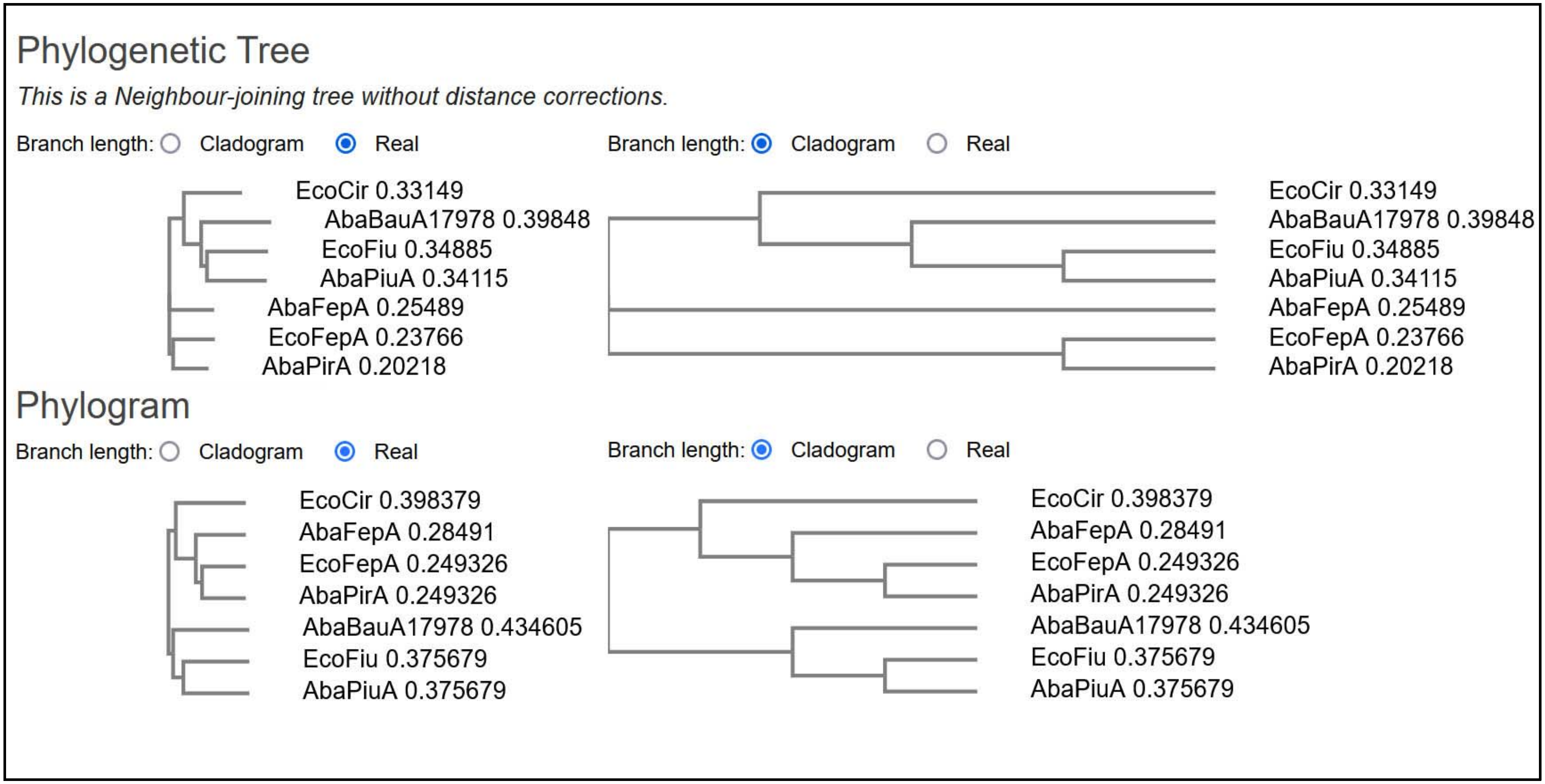

Figure S6. Sequence relationships among the ferric catecholate transporters of *E. coli* and *A. baumannii*. Output of CLUSTALω alignments of the E. coli LGP Fiu, FepA and Cir, and the A. baumannii LGP PiuA, FepA, PirA and BauA.

Video S1. Model of FeEnt* Binding and Transport by Fiu:

FiuBinding&Transport.mov
